# Tubular CD44 plays a key role in aggravating AKI through NF-κB p65-mediated mitochondrial dysfunction

**DOI:** 10.1038/s41419-025-07438-x

**Published:** 2025-02-20

**Authors:** Jiewu Huang, Ping Meng, Ye Liang, Xiaolong Li, Shan Zhou, Jiemei Li, Xiaoxu Wang, Jinhua Miao, Weiwei Shen, Lili Zhou

**Affiliations:** 1 Division of Nephrology, Nanfang Hospital, Southern Medical University, National Clinical Research Center for Kidney Disease, State Key Laboratory of Organ Failure Research, Guangdong Provincial Institute of Nephrology, Guangdong Provincial Key Laboratory of Renal Failure Research, Guangzhou, China; 2 Department of Central Laboratory, Huadu District People’s Hospital of Guangzhou, Guangzhou, China

**Keywords:** Apoptosis, Energy metabolism

## Abstract

Acute kidney injury (AKI) is in rapid prevalence nowadays. Of note, the underlying mechanisms have not been clarified. Several reports showed a cluster of differentiation-44 (CD44), a cell-surface glycoprotein, might be involved in AKI. However, its role in AKI has not been clearly clarified. Herein, we found CD44 increased in renal tubules in AKI mice. Gene ablation of CD44 improved mitochondrial biogenesis and fatty acid oxidation (FAO) function, further protecting against tubular cell death and kidney injury. Conversely, ectopic CD44 impaired mitochondrial homeostasis and exacerbated tubular cell apoptosis to aggravate AKI progression. From transcriptome sequencing, we found that CD44 induces mitogen-activated protein kinase (MAPK) and NF-κB p65 signaling. Lipidomics also showed that CD44 interfered with multiple aspects of lipid metabolism. We deeply investigated NF-κB p65 inhibited the transcription of peroxisome proliferator-activated receptor gamma coactivator 1-alpha (PGC-1α), resulting in mitochondrial dysfunction and cell apoptosis. CD44 also facilitated iron intake to assist cell ferroptosis. Hence, our study provided a new mechanism for AKI, and demonstrated that targeted inhibition on CD44 could be a promising therapeutic strategy to resist AKI.

## Introduction

AKI is characterized by rapid decline of renal function and is becoming a major public health problem all over the world [1]. The leading causes of AKI include nephrotoxins, sepsis, and especially, ischemia-reperfusion injury (IRI) [2]. Severe AKI is highly associated with cardiovascular events and strongly contributes to chronic kidney diseases (CKD), with a high mortality rate [1, 3]. Whereas, AKI is still in an awkward situation of no therapeutic strategies except renal replacement therapy, leading to a huge healthcare cost [4]. Therefore, to clarify the underlying mechanisms of AKI could provide new insight to find a targeted therapy approach.

Renal tubular epithelial cells (TECs) are the main cells in kidney parenchyma. They execute reabsorption, secretion, and excretion functions, leading to huge energy demand [2]. Hence, they are vulnerable to injury. To meet the high need for energy, the majority of TECs (proximal tubular cells) conduct fatty acid oxidation (FAO), majorly in mitochondria, to supply adenosine triphosphate (ATP) [5]. FAO deficiency would accelerate mitochondrial dysfunction to construct a vicious cycle in AKI [6]. This leads to energy deficiency, lipotoxicity, and even oxygen species (ROS) accumulation, all important triggers to induce programmed cell death [2].

CD44, a cell-surface glycoprotein, is associated with cell proliferation, migration, inflammation, wound healing, motility, cell plasticity, and metal uptake [7–11]. Our recent study found that CD44 plays an important role in CKD [12]. Others also found it is involved in AKI [3] and kidney aging [13]. In AKI, CD44 is upregulated in TECs to facilitate recruitment of leukocytes [3]. In other organs, CD44 is involved in lipid accumulation by regulating mitochondrial oxidative phosphorylation (OXPHOS) [7]. However, the role of CD44 in mitochondrial function and lipid metabolism in AKI has not been investigated in detail.

PGC-1α is a key transcription factor regulating mitochondrial biogenesis and FAO function. On one hand, PGC-1α could activate nuclear respiratory factors (NRF)1 and NRF2 to drive the transcription of mitochondrial biogenesis-related genes. On the other hand, PGC-1α interacts with peroxisome proliferator-activated receptor alpha (PPARα), the master regulator of FAO, to upregulate the expression of FAO-related genes [14]. In AKI, PGC-1α is strongly downregulated. However, the underlying mechanisms have not been elucidated.

Nuclear factor κB (NF-κB) p65 subunit, a transcriptional factor participating in many cellular processes, is greatly involved in inflammatory responses, immunity, cell migration, and apoptosis [15]. Studies in other organs showed NF-κB p65 transcriptionally silences PGC-1α promoter activity, resulting in mitochondrial dysfunction, energy insufficiency, lipid accumulation, and further cell death [16]. However, in TECs, whether PGC-1α is regulated by NF-κB p65 and its association with CD44 have not been demonstrated.

In this study, we found that CD44 was upregulated in TECs in IRI mice. CD44 further promoted the MAPK/NF-κB p65 pathway to silence transcription of PGC-1α. This highly contributed to mitochondrial dysfunction and induced TEC apoptosis and AKI. Our finding provided a new mechanism and implicated an important therapeutic strategy of targeted inhibition of CD44 to treat AKI.

## Results

### CD44 is upregulated in TECs and associated with mitochondrial dysfunction and apoptosis

**Fig. 1:**
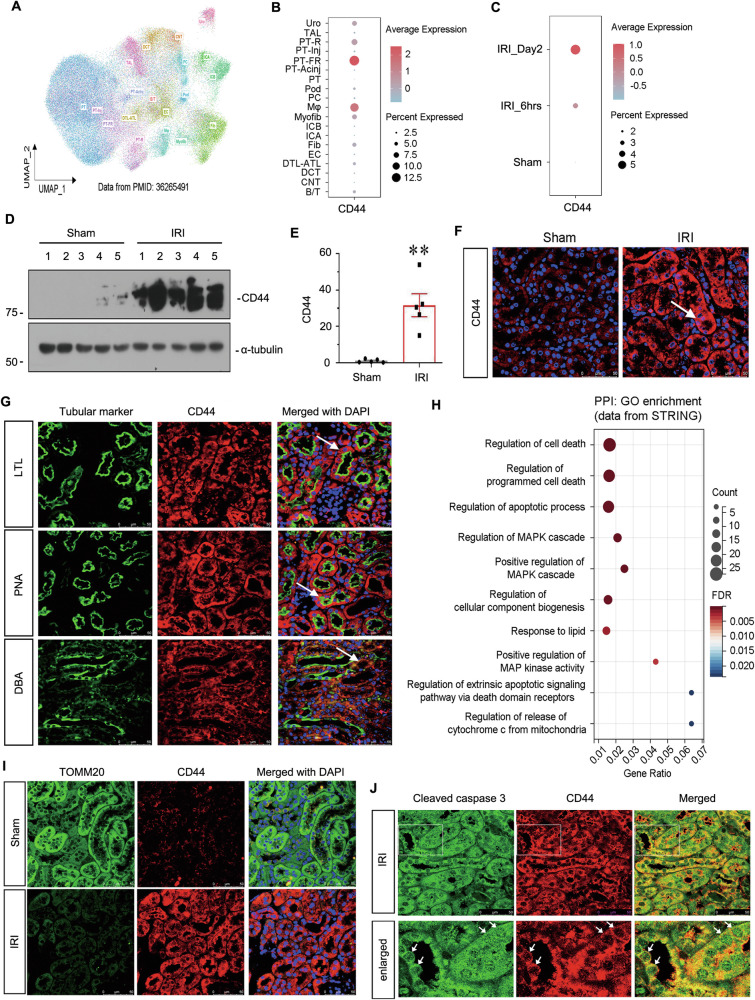
CD44 is upregulated in TECs and associated with mitochondrial dysfunction and apoptosis. **A** UMAP shows cell population in kidneys of sham and IRI at 6 h and day 2. PT proximal tubule, PT-Inj injured PT, PT-R repairing PT, FR-PTC failed repair PT cell, PT-AcInj acute injury PT, DTL descending limb of loop of Henle (LoH), ATL thin ascending limb of LoH, TAL thick ascending limb of LoH, DCT distal convoluted tubule, CNT connecting tubule, PC principal cell of collecting duct, ICA type A intercalated cell of collecting duct, ICB type B intercalated cell of collecting duct, Pod podocyte, EC endothelial cell, Fib fibroblast, Myofib myofibroblast, Ma macrophage (Mφ), B/T immune cell, Uro urothelium. Data from PMID: 36265491. **B** Graphic presentation of single-cell sequencing analysis shows the expression of CD44 in different cell populations. **C** Graphic presentation of single-cell sequencing analysis shows the expression of CD44 at different time point. **D** and **E** Representative western blot of CD44 (**D**) and graphical presentations (**E**) of protein expressional levels are shown. **P < 0.01 versus sham group (*n* = 5). **F** Representative micrographs show the expression of CD44 in sham and IRI groups, as indicated. Frozen kidney sections were stained with an antibody against CD44. Arrow indicates positive staining. Scale bar, 50 μm. **G** Co-localization staining of CD44 and various segment-specific tubular markers in the kidneys of the IRI model. Frozen kidney sections were collected from the mice 1 day after IRI. CD44 (red) and various segment-specific tubular markers (green), including LTL, PNA, and DBA, were detected by immunofluorescence. Arrows indicate positive staining. Scale bar, 50 μm. **H** GO analysis of CD44-related pathway through STRING (https://cn.string-db.org/). **I** Co-localization of CD44 and TOMM20 in tubules. Frozen renal sections were subjected to immunostaining of CD44 (red) and TOMM20 (green). Scale bar, 50 μm. **J** Co-localization of CD44 and cleaved caspase 3 in IRI group. Frozen kidney sections were subjected to immunostaining of CD44 (red) and cleaved caspase 3 (green). Scale bar, 50 μm.

To identify the role of CD44, we first evaluated the expression of CD44 in sham and AKI mice using a single-cell RNA-seq dataset (Fig. 1A) [17]. As shown in Fig. 1B, C, CD44 was mainly expressed in fail-repaired proximal tubular cells (PT-FR), in a time-independent manner. We then examined the expression of CD44 in various experimental AKI animal models, including IRI, cisplatin, and rhabdomyolysis-induced AKI. As shown, CD44 was upregulated in tubules in all three AKI models (Fig. 1D–F, Supplementary Fig. S1A–F). To further test the location of CD44, we then performed the co-staining of CD44 and different segment-specific tubular cell markers, including lotus tetragonolobus lectin (LTL), a marker of proximal tubules, peanut agglutinin (PNA), a maker of distal tubules, and dolichos biflflorus agglutinin (DBA), a marker of the collecting duct. As shown in Fig. 1G, CD44 was primarily induced in proximal and distal tubular tubules, but also with a weak increase in collecting ducts.

In order to explore the functions of CD44, we performed gene ontology (GO) enrichment analysis via protein–protein interaction (PPI) in STRING, a web for PPI. As shown in Fig. 1H, CD44 was related to the signaling pathway of regulation of MAPK, cellular apoptosis, response to lipids, biogenesis of cellular components, and mitochondria. These implicated that CD44 may be involved in cell apoptosis in AKI, and this is associated with mitochondrial injury. To test this hypothesis, we then performed the co-staining of CD44 and the translocase of outer mitochondrial membrane 20 (TOMM20), an outer mitochondrial membrane protein in sham and IRI mice. As shown in Fig. 1I, TOMM20 was highly expressed in sham mice, but largely lost in IRI mice. Inversely, CD44 was highly increased, especially in tubules in IRI mice, compared to the weak signal in sham mice. Of note, CD44 and TOMM20 have an interlaced expression mode, and their expression showed a negative correlation (Supplementary Fig. S1G). The co-staining of CD44 and cleaved caspase 3, an effector molecule in the process of apoptosis, was further assessed. As shown in Fig. 1J and Supplementary Fig. S1H, in IRI mice, both of them were upregulated in tubules. Moreover, they perfectly co-localized in the same tubular cells. Moreover, as shown in Supplementary Fig. SI, their expression showed a positive correlation. These results suggested that CD44 possibly triggered tubular cell apoptosis and is associated with the inhibition of mitochondrial function.

### Gene ablation of CD44 attenuates renal tubular cell apoptosis and kidney injury upon IRI

**Fig. 2:**
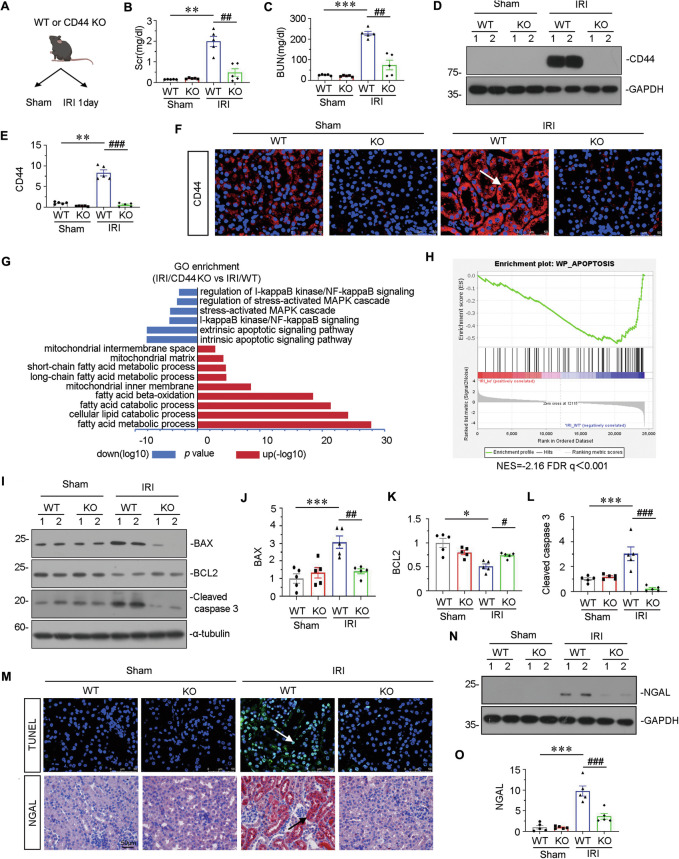
Gene ablation of CD44 attenuates renal tubular cell apoptosis and kidney injury upon IRI. **A** Experimental design: Wild-type mice and CD44 conventional knockout mice were subjected to IRI or sham, respectively, and euthanized 24 h after IRI. **B** Scr levels in four groups, as indicated. Scr was expressed as milligrams per deciliter. **P < 0.01 versus wild-type mice upon sham group; ^##^*P* < 0.01 versus wild-type mice upon IRI group (*n* = 5). **C** BUN levels in four groups, as indicated. BUN was expressed as milligrams per deciliter. ***P < 0.001 versus wild-type mice upon sham group; ^##^*P* < 0.01 versus wild-type mice upon IRI group (*n* = 5). **D** and **E** Representative western blot of CD44 (**D**) and graphical presentations (**E**) of protein expressional levels are shown. **P < 0.01 versus wild-type mice upon sham group; ^###^*P* < 0.001 versus wild-type mice upon IRI group (*n* = 5). **F** Representative micrographs show the expression of CD44 in different groups, as indicated. Frozen kidney sections were stained with an antibody against CD44. Arrow indicates positive staining. Scale bar, 50 μm. **G** GO analysis shows CD44 is involved with several important pathways, including MAPK, NF-κB, apoptosis, mitochondria and FAO. **H** GSEA shows that negative regulation of apoptosis was enriched in CD44 knockout mice versus wild-type mice upon IRI. NES, normalized enrichment score; FDR q-value < 0.25. **I–L** Representative western blot (**I**) and graphical presentations of **J** BAX, **K** BCL2, and **L** cleaved caspase 3 protein expressional levels are shown. *P < 0.05, ***P < 0.001 versus wild-type mice upon sham group; ^#^*P* < 0.05, ^##^*P* < 0.01, ^###^*P* < 0.001 versus wild-type mice upon IRI group (*n* = 5). **M** Representative micrographs show TUNEL assay in different groups, as indicated. Frozen kidney sections were subjected to TUNEL assay. Parrafin sections were performed by immunohistochemistry staining of NGAL. Arrow indicates positive staining. Scale bar, 50 μm. **N** and **O** Representative western blot of NGAL (**N**) and graphical presentations (**O**) of protein expressional levels are shown. ***P < 0.001 versus wild-type mice upon sham group; ^###^*P* < 0.001 versus wild-type mice upon IRI group (*n* = 5).

We then constructed an IRI model in both wild-type mice (WT) and CD44 knockout (KO) mice (Supplementary Fig. S1J). The experimental design is presented in Fig. 2A. Serum creatinine (Scr) and blood urea nitrogen (BUN) levels were increased in IRI mice, but both were significantly suppressed by CD44 gene knockout (Fig. 2B, C). As shown in Fig. 2D–F, CD44 expression was strongly induced in IRI mice but greatly decreased in CD44 knockout mice.

We then performed the transcriptomic sequencing analysis. As shown in Fig. 2G, gene ontology (GO) enrichment analysis of the expression of transcripts exhibited mitochondrial biogenesis, and FAO activities were activated, while apoptosis, MAPK signaling, and NF-κB p65 signaling were downregulated in CD44 knockout mice. Heatmap analysis also showed the same biological processes (Supplementary Fig. S1K). Gene Set Enrichment Analysis (GSEA) also showed that CD44 gene ablation effectively suppressed the process of apoptosis (Fig. 2H).

We next analyzed tubular cell apoptosis. Consistent with bioinformatics analysis, we found BAX and cleaved caspase 3, the pro-apoptotic proteins, were increased in IRI mice, but significantly downregulated in CD44 knockout mice. Moreover, BCL2, a pro-survival protein, was decreased in IRI mice, but significantly reversed by CD44 gene knockout (Fig. 2I–L). Furthermore, the TUNEL assay, a golden standard measure of cellular apoptosis, also showed CD44 knockout strongly inhibited tubular cell apoptosis (Fig. 2M). Similar results were observed when neutrophil gelatinase-associated lipocalin (NGAL), biomarkers of tubular cell injury, were examined by immunohistochemistry and western blotting (Fig. 2M–O). These results further demonstrated that CD44 contributed to AKI by inducing tubular cell apoptosis.

### CD44 knockout ameliorates mitochondrial dysfunction and FAO deficiency in IRI mice

**Fig. 3:**
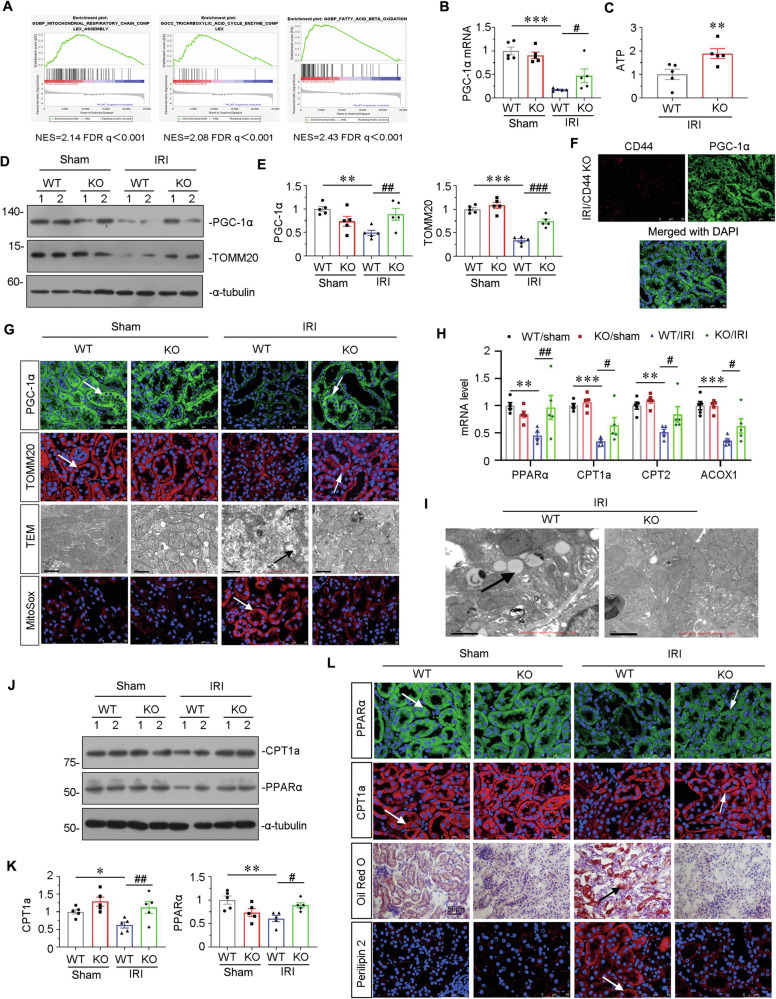
CD44 knockout ameliorates mitochondrial dysfunction and FAO deficiency in IRI mice. **A** GSEA shows that positive regulation of mitochondrial function and FAO was enriched in CD44 knockout mice versus wild-type mice upon IRI. NES, normalized enrichment score; FDR *q*-value < 0.25. **B** Graphic presentation shows the relative levels of renal expression of PGC-1α mRNA in different groups as indicated. ***P < 0.001 versus wild-type mice upon sham group; ^#^*P* < 0.05 versus wild-type mice upon IRI group (*n* = 5). **C** Graphic presentation shows the relative levels of ATP production in 2 groups as indicated. **P < 0.01 versus wild-type upon IRI group (*n* = 5). **D** and **E** Representative western blot (**D**) and graphical presentations of PGC-1α and TOMM20 protein expression levels are shown. **P < 0.01, ***P < 0.001 versus wild-type mice upon sham group; ^##^*P* < 0.01, ^###^*P* < 0.001 versus wild-type mice upon IRI group (*n* = 5). **F** Co-localization of CD44 and PGC-1α in CD44 gene ablation mice upon IRI. Frozen kidney sections were subjected to immunostaining of CD44 (red) and PGC-1α (green). Scale bar, 50 μm. **G** Representative micrographs show the expression of PGC-1α, TOMM20, mitochondrial morphology via TEM, and mitochondrial ROS via MitoSox staining in different groups, as indicated. Arrows indicate positive staining or impaired mitochondria. Scale bar, 50 or 1 μm, as indicated. **H** Graphic presentation shows the relative mRNA levels of renal expression of PPARα, CPT1, CPT2, and ACOX1 mRNA in different groups as indicated. **P < 0.01, ***P < 0.001 versus wild-type mice upon sham group; ^#^*P* < 0.05, ^##^*P* < 0.01 versus wild-type mice upon IRI group (*n* = 5). **I** Representative micrographs show the abundance of LDs via TEM in 2 groups, as indicated. Arrows indicate LDs. Scale bar, 1 μm. **J** and **K** Representative western blot (**J**) and graphical presentations of CPT1a and PPARα protein expression levels are shown. *P < 0.05, **P < 0.01 versus wild-type mice upon sham group; ^#^*P* < 0.05, ^##^*P* < 0.01 versus wild-type mice upon IRI group (*n* = 5). **L** Representative micrographs show the expression of PPARα, CPT1a, perilipin 2, and LDs via Oil Red O staining in different groups, as indicated. Arrows indicate positive staining. Frozen kidney sections were subjected to Oil Red O staining or stained with antibodies against PPARα, CPT1a, and perilipin 2. Scale bar, 50 μm.

We then assessed mitochondrial function and lipid metabolism. GSEA analysis was first performed and revealed that CD44 knockout effectively restored mitochondrial respiratory, tricarboxylic acid cycle (TCA), and FAO function (Fig. 3A). We then tested PGC-1α mRNA levels and ATP production. As shown in Fig. 3B, C, the expression of PGC-1α mRNA was downregulated in IRI mice but significantly reversed after CD44 gene ablation. Consistently, ATP production was also largely preserved in CD44 knockout mice (Fig. 3C). Similar results were observed when PGC-1α and TOMM20, the markers for mitochondrial biogenesis, were assessed by western blotting and immunofluorescence (Fig. 3D, E, G). Of note, we observed the staggered expression of CD44 and PGC-1α, further suggesting the negative regulation between them (Fig. 3F). To further analyze mitochondrial function, we performed transmission electron microscope (TEM) and mitoSox staining to check mitochondrial morphology and mitochondrial ROS production. As shown in Fig. 3G, IRI-induced mitochondrial damages, characterized by swelling, fragmented cristae, and decrease in numbers, and mitochondrial ROS production were greatly inhibited by CD44 knockout.

**Fig. 4:**
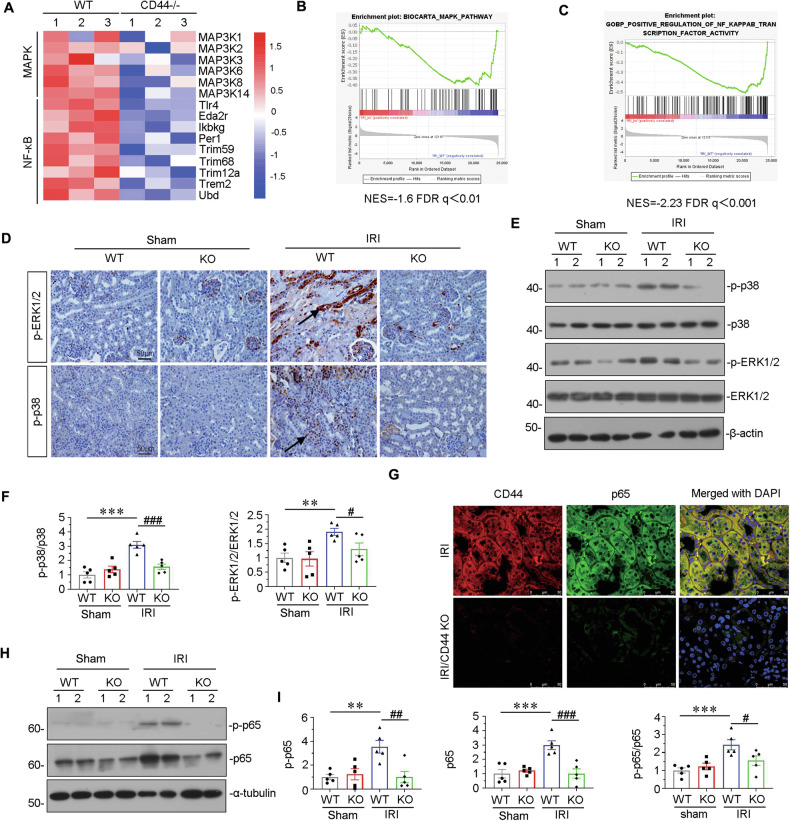
CD44 promotes AKI progression through inducing MAPK and NF-κB p65 signaling. **A** Representative heatmap gene expression of RNA sequencing analysis shows that CD44 is involved with MAPK and NF-κB signaling pathway. **B** and **C** GSEA shows that negative regulation of MAPK and NF-κB pathway was enriched in CD44 knockout mice versus wild-type mice upon IRI. NES, normalized enrichment score; FDR *q*-value < 0.25. **D** Representative micrographs show the expression of p-ERK1/2 and p-p38 in different groups, as indicated. Paraffin sections were stained with antibodies against p-ERK1/2 and p-p38. Arrows indicate positive staining. Scale bar, 50 μm. **E** and **F** Representative western blot (**E**) and graphical presentations of p-p38/p38 and p-ERK1/2/ERK1/2 protein levels are shown. **P < 0.01, ***P < 0.001 versus wild-type mice upon sham group; ^#^*P* < 0.05, ^###^*P* < 0.001 versus wild-type mice upon IRI group (*n* = 5). **G** Co-localization of CD44 and p65 in 2 groups, as indicated. Frozen kidney sections were subjected to immunostaining of CD44 (red) and p65 (green). Scale bar, 50 μm. **H** and **I** Representative western blot (**H**) and graphical presentations of p-p65, p65, and p-p65/p65 protein levels are shown. **P < 0.01, ***P < 0.001 versus wild-type mice upon sham group; ^#^*P* < 0.05, ^##^*P* < 0.01, ^###^*P* < 0.001 versus wild-type mice upon IRI group (*n* = 5).

We next assessed FAO and lipid metabolism. As shown in Fig. 3H, CD44 knockout restored the mRNA expression of PPARα, carnitine palmitoyl transferase 1A (CPT1a), carnitine palmitoyltransferase 2 (CPT2), and acyl-coenzyme A oxidase 1 (ACOX1), the rate-limiting enzymes controlling the key steps of FAO. TEM analysis also revealed IRI induced an evident accumulation of lipid droplets in proximal tubular cells, while CD44 knockout strongly inhibited it (Fig. 3I). Western blotting also showed CPT1a and PPARα were greatly restored in CD44 KO mice (Fig. 3J, K). Similar results were observed when CPT1a and PPARα were assessed by immunofluoresence (Fig. 3L). To further identify the role of CD44 in lipid metabolism, we performed Oil Red O staining (for the detection of neutral lipids) and examined the expression of lipid droplet-associated protein perilipin 2. As shown in Fig. 3L, large amounts of lipid accumulated in the IRI-affected kidney, while not in the kidney of CD44 KO mice. These results further suggest that CD44 plays a role in mitochondrial dysfunction and lipid metabolism abnormality in renal tubular cells.

### CD44 promotes AKI progression through inducing MAPK and NF-κB p65 signaling

From transcriptomic sequencing analysis, we found MAPK and NF-κB p65 signaling pathways were involved in CD44 signaling (Fig. 4A). To confirm it, cultured HKC-8 cells were transfected with CD44 expression plasmid. As shown, overexpression of CD44 induced ERK1/2 and p38 signaling pathways, but not JNK (Supplementary Fig. S1L and M), and further triggered the activation of NF-κB p65 (Supplementary Fig. S1L and M).

We then performed GSEA analysis and found that CD44 knockout indeed inhibited MAPK and NF-κB p65 signaling pathways (Fig. 4B, C). ERK1/2 and p38 signaling pathways were examined in IRI mice. As shown, IRI induces phosphorylation of ERK1/2 and p38, but CD44 knockout blocked it (Fig. 4D). Similar results were observed when they were examined by the western bolt (Fig. 4E, F). NF-κB p65 signaling was then examined. As shown, CD44 and NF-κB p65 were upregulated in IRI mice, but significantly inhibited by CD44 KO (Fig. 4G). Moreover, the expression of CD44 was positively correlated with NF-κB p65 (Supplementary Fig. S1N). Similarly, CD44 and phosphorylated NF-κB p65 (the active form of p65) were activated in IRI mice but significantly inhibited by CD44 knockout, when p-p65 and p65 were assessed by western blotting (Fig. 4H, I). These results further suggested CD44 induced MAPK-NF-κB p65 signaling to aggravate AKI.

### Ectopic CD44 aggravates tubular cell apoptosis and kidney injury in IRI mice

**Fig. 5:**
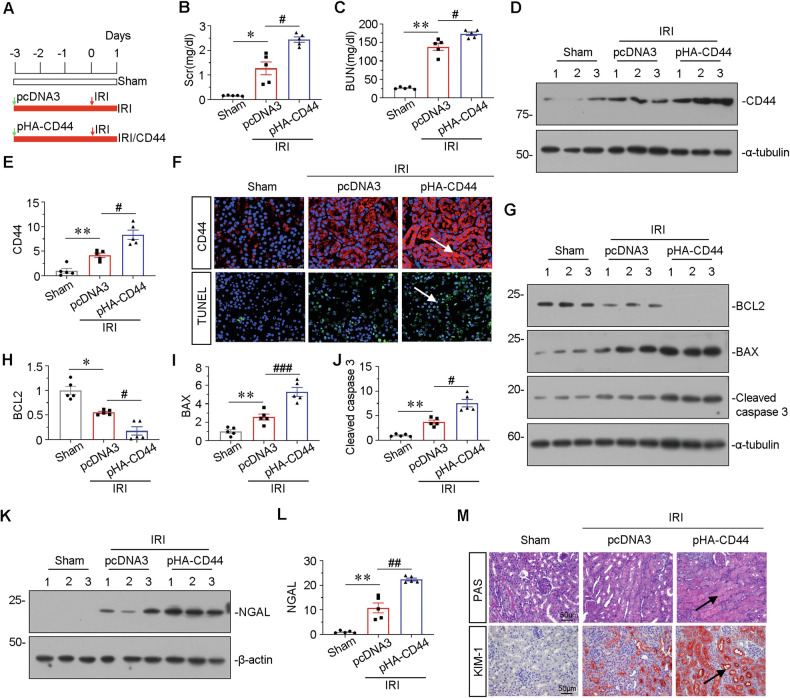
Ectopic CD44 aggravates tubular cell apoptosis and kidney injury in IRI mice. **A** Experimental design: Green arrow indicated the injection of pcDNA3 plasmid or p-HA-CD44 overexpression plasmid. Mice were subjected to IRI surgery or sham surgery, respectively, as shown in the red arrow. Mice are euthanized 24 h after surgery. **B** Scr levels in three groups, as indicated. Scr was expressed as milligrams per deciliter. *P < 0.05 versus sham group; ^#^*P* < 0.05 versus IRI group injected with pcDNA3 (*n* = 5). **C** BUN levels in three groups, as indicated. BUN was expressed as milligrams per deciliter. **P < 0.01 versus sham group; ^#^*P* < 0.05 versus IRI group injected with pcDNA3 (*n* = 5). **D** and **E** Representative western blot of CD44 (**D**) and graphical presentations (**E**) of protein levels are shown. **P < 0.01 versus sham group; ^#^*P* < 0.05 versus IRI group injected with pcDNA3 (*n* = 5). **F** Representative micrographs show the expression of CD44, and TUNEL assay in different groups, as indicated. Arrows indicate positive staining. Frozen kidney sections were subjected to TUNEL assay or stained with an antibody against CD44. Scale bar, 50 μm. **G–J** Representative western blot (**G**) and graphical presentations of **H** BCL2, **I** BAX, and **J** cleaved caspase 3 protein expression levels are shown. *P < 0.05, **P < 0.01 versus sham group; ^#^*P* < 0.05, ^###^*P* < 0.001 versus IRI group injected with pcDNA3 (*n* = 5). **K** and **L** Representative western blot of NGAL (**K**) and graphical presentations (**L**) of protein levels are shown. **P < 0.01 versus sham group; ^##^*P* < 0.01 versus IRI group injected with pcDNA3 (*n* = 5). **M** Representative micrographs show renal tubular morphologic injury and the expression of KIM-1 in different groups, as indicated. Paraffin sections were subjected to PAS staining, and were stained with an antibody against KIM-1. Arrows indicate positive staining. Scale bar, 50 μm.

To further identify the role of CD44 in IRI, we delivered CD44 expression plasmid into IRI mice through hydrodynamic-based delivery, a common approach to express proteins in the kidney [18]. The experimental design is shown in Fig. 5A. As shown in Fig. 5B, C, ectopic expression of CD44 further elevated the levels of Scr and BUN. CD44 expression was assessed by western blot and immunofluorescence (Fig. 5D–F) and exhibited further induction by delivery of CD44 expression plasmid.

We then assessed tubular cell apoptosis and injury. As shown in Fig. 5F, TUNEL assay revealed IRI-induced tubular cell apoptosis, which was further aggravated by ectopic expression of CD44. Furthermore, apoptosis-related proteins were analyzed by western blotting. As shown, overexpression of CD44 further decreased the expression of BCL2 and triggered the upregulation of BAX and cleaved caspase 3 (Fig. 5G–J). Tubular cell injury was also assessed. As shown, ectopic CD44 further promotes the expression of NGAL and kidney injury molecule 1 (KIM-1) (Fig. 5K–M). Periodic acid–Schiff (PAS) staining also evidently demonstrated tubular cell damages, including tubular dilation, cell detachment, and cast formation, which were further aggravated by ectopic CD44 in IRI mice (Fig. 5M).

### Ectopic expression of CD44 impairs mitochondrial function and FAO through activating MAPK and NF-κB p65 signaling

**Fig. 6:**
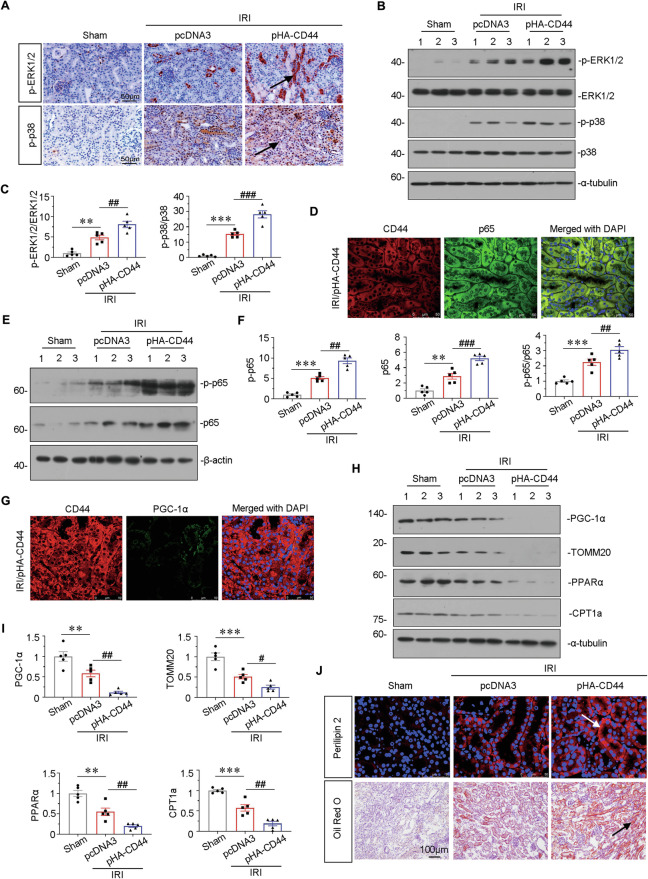
Ectopic expression of CD44 impairs mitochondrial function and FAO through activating MAPK and NF-κB p65 signaling. **A** Representative micrographs show the expression of p-ERK1/2 and p-p38 in different groups, as indicated. Paraffin sections were stained with antibodies against p-ERK1/2 and p-p38. Arrows indicate positive staining. Scale bar, 50 μm. **B** and **C** Representative western blot (**B**) and graphical presentations of p-ERK1/2/ERK1/2 and p-p38/p38 protein levels are shown. **P < 0.01, ***P < 0.001 versus sham group; ^##^*P* < 0.01, ^###^*P* < 0.001 versus IRI group injected with pcDNA3 (*n* = 5). **D** Co-localization of CD44 and p65 in CD44 overexpression mice upon IRI. Frozen renal sections were subjected to immunostaining of CD44 (red) and p65 (green). Scale bar, 50 μm. **E** and **F** Representative western blot (**E**) and graphical presentations of p-p65, p65, and p-p65/p65 protein levels are shown. **P < 0.01, ***P < 0.001 versus sham group; ^##^*P* < 0.01, ^###^*P* < 0.001 versus IRI group injected with pcDNA3 (*n* = 5). **G** Co-localization of CD44 and PGC-1α in CD44 overexpression mice upon IRI. Frozen renal sections were subjected to immunostaining of CD44 (red) and PGC-1α (green). Scale bar, 50 μm. **H** and **I** Representative western blot (**H**) and graphical presentations of PGC-1α, TOMM20, PPARα, and CPT1a protein expression levels are shown. **P < 0.01, ***P < 0.001 versus sham group; ^#^*P* < 0.05, ^##^*P* < 0.01 versus IRI group injected with pcDNA3 (*n* = 5). **J** Representative micrographs show the expression of perilipin 2, and LDs via Oil Red O staining in different groups, as indicated. Frozen kidney sections were subjected to Oil Red O staining or stained with an antibody against perilipin 2. Arrows indicate positive staining. Scale bar, 50 or 100 μm.

As shown in Fig. 6A–C, ectopic expression of CD44 greatly induced the upregulation of phosphorylated ERK1/2 and p38, the active forms of those kinases. Moreover, as shown in Fig. 6D, co-staining of CD44 and p65 implied that CD44 is positively related to the activation of NF-κB p65. Western blotting also showed ectopic CD44 further induced the phosphorylation and activation of NF-κB p65 (Fig. 6E, F).

Mitochondrial function and lipid metabolism were then assessed. As shown in Fig. 6G, ectopic expression of CD44 triggered the loss of PGC-1α. Western blotting and immunofluorescence showed ectopic CD44 further exaggerated the loss of PGC-1 and TOMM20, two markers of mitochondrial biogenesis. Furthermore, overexpression of CD44 also accelerated the downregulation of PPARα and CPT1a, two key regulators controlling FAO function, in IRI mice (Fig. 6H, I and Supplementary Fig. S1O). Similarly, Oil Red O and perilipin 2 staining showed ectopic CD44 further promoted lipid accumulation in renal tubules in IRI mice (Fig. 6J). These results further demonstrated that CD44 aggravated AKI through disrupting mitochondrial function and lipid metabolism.

### CD44 aggravates mitochondrial and FAO dysfunction, and drives cell apoptosis though MAPK and NF-κB p65 signaling in vitro

**Fig. 7:**
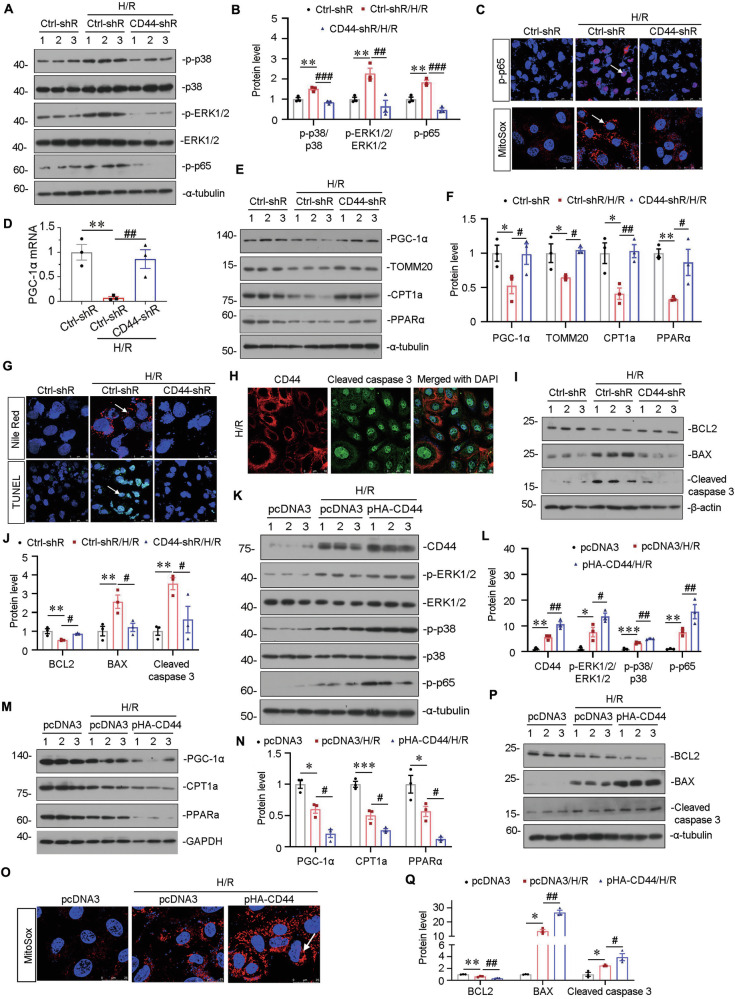
CD44 aggravates mitochondrial and FAO dysfunction and drives cell apoptosis through MAPK and NF-κB p65 signaling in vitro. **A** and **B** HKC-8 was transfected with Ctrl-shR or CD44-shR and then were incubated in basal culture medium in a 1% O_2_ environment for 24 h and then were reoxygenated in normal O_2_ for 6 h. Representative western blot (**A**) and graphical presentations of p-p38/p38, p-ERK1/2/ERK1/2, and p-p65 protein expression levels are shown. **P < 0.01 versus Crtl-shR group; ^##^*P* < 0.01, ^###^*P* < 0.001 versus H/R with Crtl-shR group (*n* = 3). **C** Representative micrographs show the expression of p-p65 and MitoSox staining in different groups, as indicated. Arrows indicate positive staining. Cells cultured on coverslips were stained with an antibody against p-p65 or were stained with MitoSox. Scale bar, 25 or 50 μm. **D** Graphic presentation shows the relative mRNA levels of PGC-1α in different groups as indicated. **P < 0.01 versus Crtl-shR group; ^##^*P* < 0.01 versus H/R with Crtl-shR group (*n* = 3). **E** and **F** Representative western blot (**E**) and graphical representations of PGC-1α, TOMM20, CPT1a, and PPARα protein expression levels are shown. *P < 0.05, **P < 0.01 versus Crtl-shR group; ^#^*P* < 0.05, ^##^*P* < 0.01 versus H/R with Crtl-shR group (*n* = 3). **G** Representative micrographs show Nile Red staining and TUNEL assay in different groups, as indicated. Arrows indicate positive staining. Cells cultured on coverships were stained with Nile Red or TUNEL assay. Scale bar, 25 or 50 μm. **H** Co-localization of CD44 and cleaved caspase 3 in HKC-8 after H/R treatment. Cells cultured on coverships were subjected to immunostaining of CD44 (red) and cleaved caspase 3 (green). Scale bar, 50 μm. **I** and **J** Representative western blot (**I**) and graphical representations of BCL2, BAX, and cleaved caspase 3 protein expression levels are shown. **P < 0.01 versus Crtl-shR group; ^#^*P* < 0.05 versus H/R with Crtl-shR group (*n* = 3). **K** and **L** HKC-8 was transfected with pcDNA3 or p-HA-CD44 overexpression plasmid and then were incubated in basal culture medium in a 1% O_2_ environment for 24 h and then were reoxygenated in normal O_2_ for 6 h. Representative western blot (**K**) and graphical representations of CD44, p-ERK1/2/ERK1/2, p-p38/p38 and p-p65 protein expression levels are shown. *P < 0.05, **P < 0.01, ***P < 0.001 versus pcDNA3 group; ^#^*P* < 0.05, ^##^*P* < 0.01 versus H/R with pcDNA3 group (*n* = 3). **M** and **N** Representative western blot (**M**) and graphical presentations of PGC-1α, CPT1a, and PPARα protein expression levels are shown. *P < 0.05, ***P < 0.001 versus pcDNA3 group; ^#^*P* < 0.05 versus H/R with pcDNA3 group (*n* = 3). **O** Representative micrographs show MitoSox staining in different groups, as indicated. Arrow indicates positive staining. Cells cultured on coverships were stained with MitoSox. Scale bar, 25 μm. **P** and **Q** Representative western blot (**P**) and graphical representations of BCL2, BAX, and cleaved caspase 3 protein expression levels are shown. *P < 0.05, **P < 0.01 versus pcDNA3 group; ^#^*P* < 0.05; ^##^*P* < 0.01 versus H/R with pcDNA3 group (*n* = 3).

To further explore the role of CD44 in cellular apoptosis, HKC-8 cells were transfected with a shRNA plasmid to interfere with CD44 under the condition of hypoxia/reoxygenation (H/R). The knockdown of CD44 is verified via QPCR in Supplementary Fig 1P. As shown in Fig. 7A–C, H/R triggered the activation of ERK1/2, p38, and p65, while knockdown of CD44 largely inhibited these effects. We further found knockdown of CD44 blocked the production of mitochondrial ROS (Fig. 7C), suggesting the negative regulation of CD44 in mitochondrial function.

We next assessed the expression of PGC-1α, TOMM20, PPARα, and CPT1a, the key players in mitochondrial biogenesis and FAO function. As shown, all of them were downregulated in the H/R group, while CD44 knockdown restored their expression (Fig. 7D–F). Furthermore, TUNEL and Nile Red staining showed that H/R induced lipid accumulation and cell apoptosis, while CD44 knockdown inhibited these effects (Fig. 7G). We also performed the double staining of CD44 and cleaved caspase 3 and found in the H/R group, they were activated concomitantly and positively correlated (Fig. 7H, Supplementary Fig. S2A). Moreover, CD44 knockdown blocked H/R-triggered downregulation of BCL2, and upregulation of cleaved caspase 3 and BAX (Fig. 7I, J).

HKC-8 cells were also transfected with CD44 expression plasmid. As shown, ectopic CD44 further aggravated the activation of ERK1/2, p38, and p65 (Fig. 7K, L), inhibited the expression of PGC-1α, TOMM20, PPARα, and CPT1a, and promoted the mitochondrial ROS production (Fig. 7M–O, Supplementary Fig. S2B and C). As a result, ectopic CD44 further decreased BCL2, increased BAX and cleaved caspase 3 expression (Fig. 7P, Q). These results further suggested that CD44 promoted mitochondrial dysfunction and tubular cell apoptosis by inducing MAPK-NF-κB p65 signaling.

### CD44 induces tubular cell injury through MAPK-NF-κB p65-silenced PGC-1α signaling

**Fig. 8:**
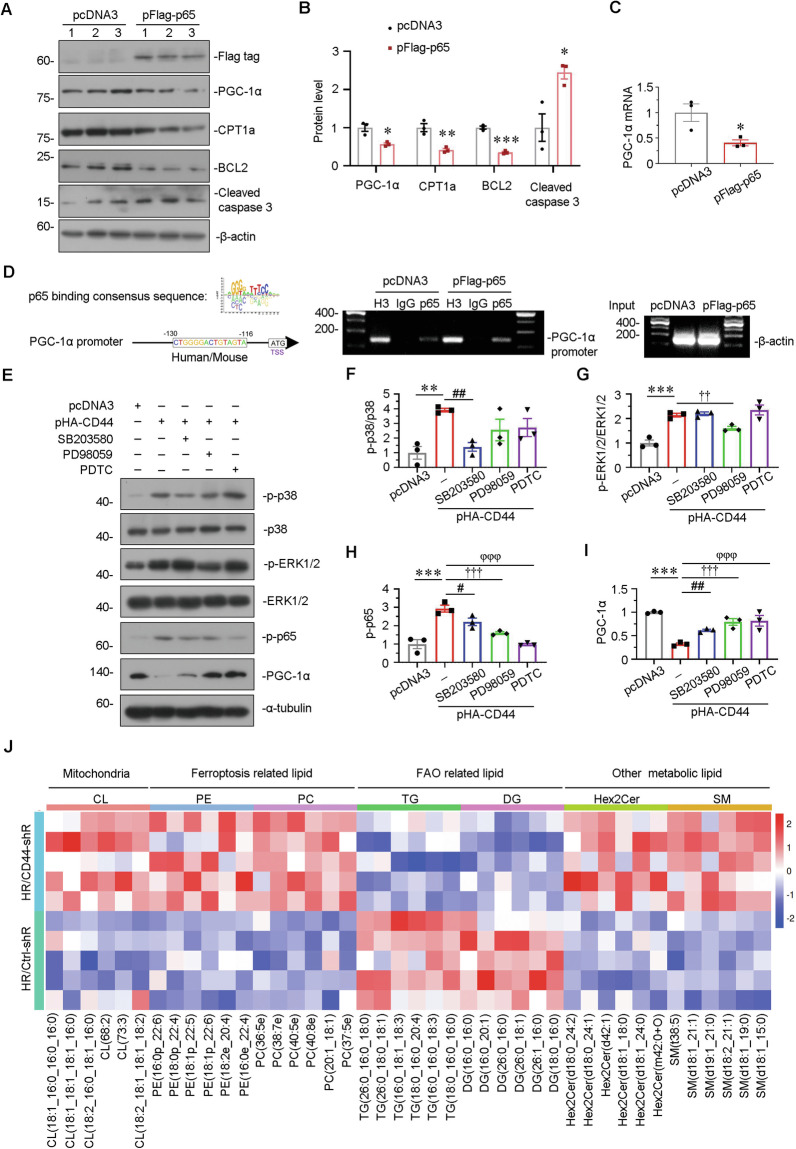
CD44 induces tubular cell injury through MAPK-NF-κB p65-silenced PGC-1α signaling. **A** and **B** HKC-8 was transfected with pcDNA3 or p-Flag-p65 overexpression plasmid for 24 h. Representative western blot (**A**) and graphical presentations of PGC-1α, CPT1a, BCL2, and cleaved caspase 3 protein expression levels are shown. *P < 0.05, **P < 0.01, ***P < 0.001 versus pcDNA3 groups (*n* = 3). **C** Quantitative PCR result showing relative mRNA level of PGC-1α. *P < 0.05 versus pcDNA3 groups (*n* = 3). **D** Representative ChIP assay results showing the binding of p65 to PGC-1α gene promoter region. HKC-8 cells were transfected with pcDNA3 or p-Flag-p65 for 24 h. Cell lysates were precipitated with an antibody against p65, histone H3, or nonimmune IgG, and ChIP assay was performed for PGC-1α gene promoters. Total diluted lysate was used as total genomic input DNA. **E–I** HKC-8 was pre-treated with SB203580, PD98059 or PDTC at 1 h before transfection with pcDNA3 or p-HA-CD44 plasmid for 24 h. Representative western blot (**E**) and graphical presentations of **F** p-p38/p38, **G** p-ERK1/2/ERK1/2, **H** p-p65, and **I** PGC-1α protein expression levels are shown. **P < 0.01, ***P < 0.001 versus pcDNA3 group; ^#^*P* < 0.05, ^##^*P* < 0.01 versus pHA-CD44 group; ^††^*P* < 0.01, ^†††^*P* < 0.001 versus pHA-CD44 group; ^φφφ^*P* < 0.001 versus pHA-CD44 group (*n* = 3). **J** The heatmap exhibiting differentiated lipids of lipidomics sequencing between H/R with Crtl-shR group and H/R with CD44-shR group.

HKC-8 was first transfected with p65 expression plasmid. As shown in Fig. 8A, B, overexpression of NF-κB p65 inhibited the protein expression of PGC-1α, CPT1a, and BCL2, but upregulated cleaved caspase 3. We further found overexpression of NF-κB p65 effectively decreased PGC-1α mRNA level (Fig. 8C), which is consistent with the other study of myocardial cells [16]. To identify the mechanisms of NF-κB p65 inhibiting PGC-1α transcription, we then performed bioinformatics analysis to check whether the PGC-1α gene promoter possesses the binding site of NF-κB p65. As shown, there is a perfect binding site of NF-κB p65 in the PGC-1α gene promoter region (Fig. 8D). Chromatin immunoprecipitation (ChIP) assay was further performed and verified the binding of NF-κB p65 with PGC-1α gene promoter.

HKC-8 cells were then treated with MAPK p38 signaling inhibitor SB203580, MAPK ERK1/2 signaling inhibitor PD98059, and NF-κB signaling inhibitor pyrrolidine dithiocarbamate (PDTC) before transfection with CD44 expression plasmid. As shown in Fig. 8E–I, ectopic CD44 activated phosphorylated activation of ERK1/2, p38, and NF-κB p65. Co-treatment with SB203580 or PD98059 could suppress NF-κB p65 phosphorylation and reverse the expression of PGC-1α. Of note, PD98059 showed more effects in CD44-overexpressed cells, suggesting ERK1/2 signaling is more significant in signaling transduction of CD44. In addition, PDTC evidently restored PGC-1α expression. These results further suggest CD44 inhibited PGC-1α through ERK1/2/NF-κB p65 pathway.

In order to explore the effects of CD44 on lipid compositions, we conducted lipidomics sequencing in HKC-8 cells. As shown in Fig. 8J, compared to H/R simulation alone, CD44 knockdown increased the content of cardiolipin, a mitochondrial signature phospholipid, which is oxidized and downregulated in dysfunctional mitochondria [19]. Furthermore, CD44 knockdown decreased triglyceride and diglyceride contents, which are accumulated during FAO dysfunction. Moreover, CD44 knockdown restored the content of phosphatidylethanolamine (PE) and phosphatidylcholine (PC), the main membrane phospholipids with peroxidation of polyunsaturated fatty acids (PUFAs), which is oxidized and degraded during the progress of ferroptosis, suggesting the role of CD44 in ferroptosis. Interestingly, CD44 knockdown also regulated the contents of sphingomyelin (SM) and dihexosylceramides (Hex2cer), and so on, suggesting CD44 affects the whole processes of lipid metabolism.

### CD44 promotes tubular cell injury and AKI partially through ferroptosis

**Fig. 9:**
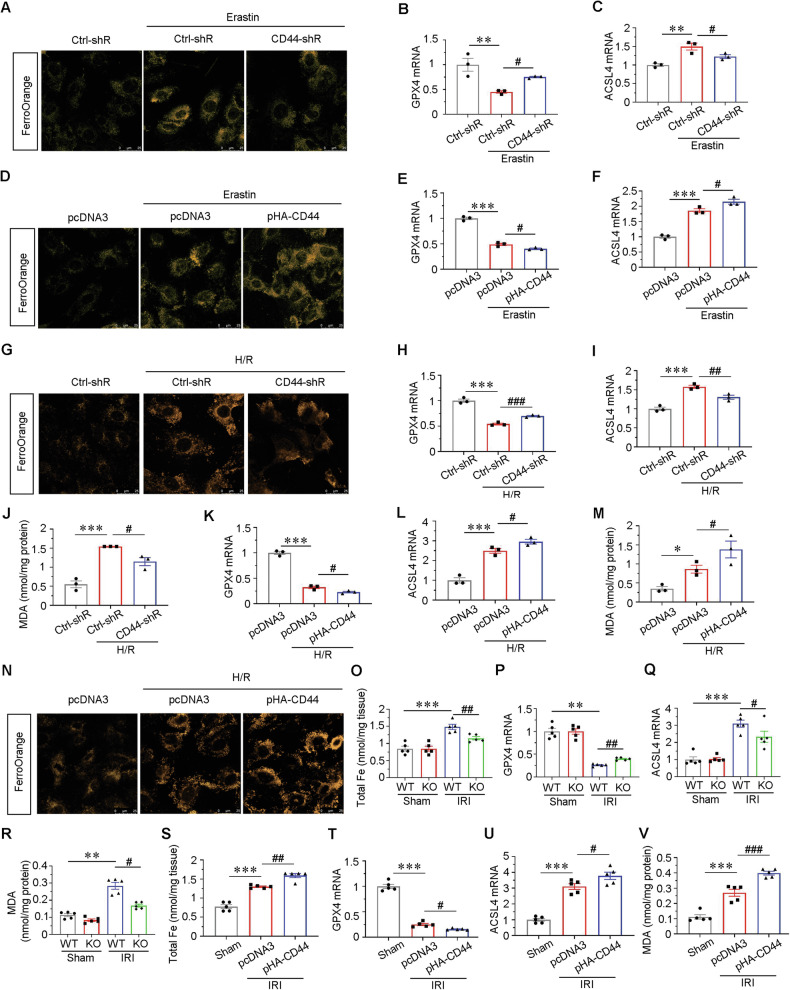
CD44 promotes tubular cell injury and AKI partially through ferroptosis. **A** HKC-8 was transfected with Ctrl-shR or CD44-shR plasmid and then treated with 5 μM erastin for 24 h. Representative micrographs show the Fe^2+^ content via FerroOrange staining in different groups, as indicated. Scale bar, 25 μm. **B** and **C** Quantitative results of QPCR showing relative (**B**) GPX4 and (**C**) ACSL4 mRNA levels among different groups. **P < 0.01 versus Ctrl-shR group; ^#^*P* < 0.05 versus erastin with ctrl-shR (*n* = 3). **D** HKC-8 was transfected with pcDNA3 or pHA-CD44 overexpression plasmid and then treated with 5 μM erastin for 24 h. Representative micrographs show the Fe^2+^ content via FerroOrange staining in different groups, as indicated. Scale bar, 25 μm. **E** and **F** Quantitative result of QPCR showing relative (**E**) GPX4 and (**F**) ACSL4 mRNA levels among different groups. ***P < 0.001 versus pcDNA3 group; ^#^*P* < 0.05 versus erastin with pcDNA3 (*n* = 3). **G** Representative micrographs show the Fe^2+^ content via FerroOrange staining in different groups, as indicated. Scale bar, 25 μm. **H** and **I** Quantitative result of QPCR showing relative **H** GPX4 and **I** ACSL4 mRNA level among different groups. ***P < 0.001 versus Ctrl-shR group; ^##^*P* < 0.01, ^###^*P* < 0.001 versus H/R with ctrl-shR (*n* = 3). **J** Quantitative result showing MDA content among different groups. ***P < 0.001 versus Ctrl-shR group; ^#^*P* < 0.05 versus H/R with ctrl-shR (*n* = 3). **K** and **L** Quantitative result of QPCR showing relative (**K**) GPX4 and (**L**) ACSL4 mRNA levels among different groups. ***P < 0.001 versus pcDNA3 group; ^#^*P* < 0.05 versus H/R with pcDNA3 (*n* = 3). **M** Quantitative result showing MDA content among different groups. ***P < 0.001 versus pcDNA3 group; ^#^*P* < 0.05 versus H/R with pcDNA3 (*n* = 3). **N** Representative micrographs show the Fe^2+^ content via FerroOrange staining in different groups, as indicated. Scale bar, 25 μm. **O** Quantitative result showing total iron content in kidney tissue among different groups. ***P < 0.001 versus wild-type mice upon sham group; ^##^*P* < 0.01 versus wild-type mice upon IRI group (*n* = 5). **P** and **Q** Quantitative result of QPCR showing relative **P** GPX4 and **Q** ACSL4 mRNA levels among different groups. **P < 0.01, ***P < 0.001 versus wild-type mice upon sham group; ^#^*P* < 0.05, ^##^*P* < 0.01 versus wild-type mice upon IRI group (*n* = 5). **R** Quantitative result showing MDA content in kidney tissue among different groups. **P < 0.01 versus wild-type mice upon sham group; ^#^*P* < 0.05 versus wild-type mice upon IRI group (*n* = 5). **S** Quantitative result showing total iron content in kidney tissue among different groups. ***P < 0.001 versus sham group; ^##^*P* < 0.01 versus IRI group injected with pcDNA3 (*n* = 5). **T** and **U** Quantitative result of QPCR showing relative **S** GPX4 and **T** ACSL4 mRNA level among different groups. ***P < 0.001 versus sham group; ^#^*P* < 0.05 versus IRI group injected with pcDNA3 (*n* = 5). **V** Quantitative result showing MDA content in kidney tissue among different groups. ***P < 0.001 versus sham group; ^###^*P* < 0.001 versus IRI group injected with pcDNA3 (*n* = 5).

Due to the effect of CD44 on ferroptosis-related lipid metabolites, we thought CD44 may also play a role in tubular cell ferroptosis. Recent studies showed CD44 may facilitate the endosomal uptake of Fe^3+^, which transfers to Fe^2+^ in endolysosomes [11] in other organs. Hence, we first assessed the Fe^2+^ content in cultured cells. FerroOrange staining showed erastin-induced uptake of Fe^2+^, however, interference of CD44 inhibited it (Fig. 9A). We further found erastin triggered the downregulation of GPX4 and upregulation of ACSL4, but these effects were reversed by CD44 knockdown to some extent (Fig. 9B, C). Inversely, overexpression of CD44 further aggravated the accumulation of Fe^2+^ and decreased GPX4, and upregulated ACSL4 (Fig. 9D–F).

We also performed studies in H/R-stimulated cells. As shown, knockdown of CD44 significantly blocked Fe^2+^ intake, restored GPX4 expression, and inhibited ACSL4 expression and reduced the content of MDA (Fig. 9G–J) in H/R-stimulated cells to some extent. Conversely, ectopic expression of CD44 further inhibited GPX4, increased ACSL4, and promoted the production of MDA, and aggravated Fe^2+^ accumulation (Fig. 9K–N).

We next explored the effects of CD44 on ferroptosis in vivo. As shown in Fig. 9O, CD44 gene ablation reduced the accumulation of iron in IRI, slightly upregulated GPX4 mRNA level, and downregulated ACSL4 mRNA level and reduced the content of MDA to some extent (Fig. 9P–R). On the contrary, ectopic CD44 further promoted iron accumulation, decreased GPX4, and increased ACSL4 and MDA production slightly (Fig. 9S–V).

Several reports also showed that CD44 plays a role in the intake of Cu^2+^ [10], suggesting CD44 may play a role in cuproptosis. To testify it, we assessed cuproptosis in vitro and in vivo. However, although CD44 gene knockdown inhibited the intake of Cu^2+^, it showed no evident effects on the mRNA levels of cuproptosis-related gene FDX1 and LIAS (Supplementary Fig. S2D–F). Furthermore, ectopic CD44 expression slightly promoted the accumulation of Cu^2+^, but it has no statistical differences in the mRNA level of FDX1 and LIAS (Supplementary Fig. S2G–I). Consistently, similar results were found in vivo. Ectopic CD44 did not affect FDX1 and LIAS expression although it slightly increased the intake of Cu^2+^ (Supplementary Fig. S2J–O).

### Schematic diagram

**Fig. 10:**
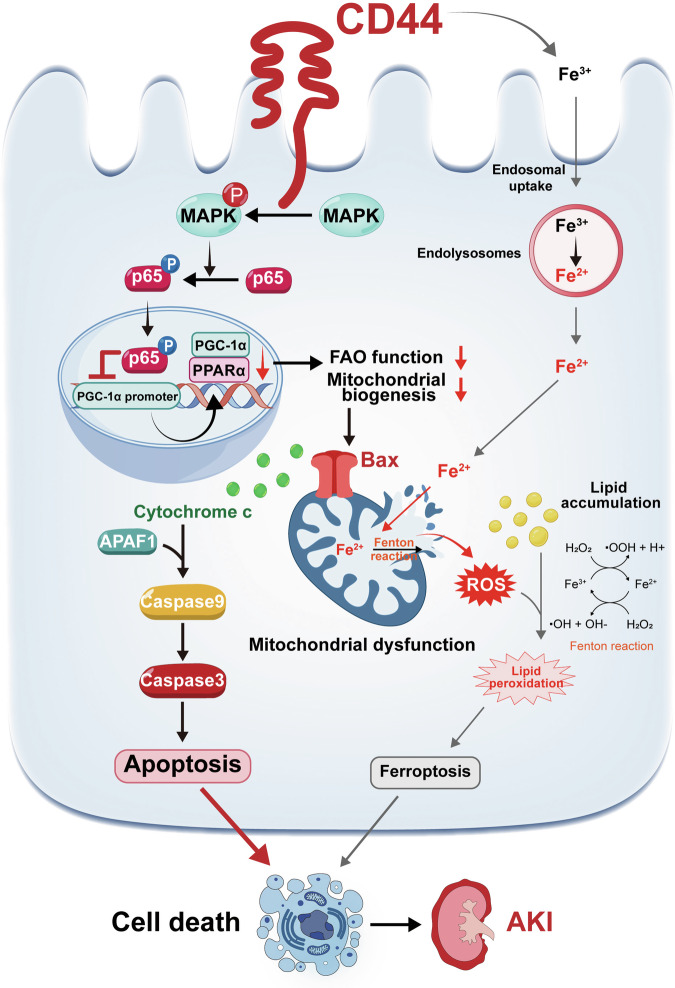
Schematic diagram. CD44 plays an important role in the pathogenesis of AKI. Through the activation of MAPK and NF-κB p65, CD44 inhibits PGC-1α transcription and subsequent binding of PGC-1α and PPARα. This leads to FAO deficiency and loss of mitochondrial biogenesis, causing lipid accumulation and mitochondrial dysfunction. Concomitantly, Bax migrates to the outer membrane of mitochondria and drives high permeability in mitochondria, resulting in the release of cytochrome c into cytoplasm. Cytochrome c then binds with apoptosis-protease-activating factor 1 (APAF1), inducing the activation cascade of caspases, such as caspase 9 and caspase 3, which finally drives tubular cellular apoptosis and initiates AKI. Additionally, CD44 also facilitates endolysosomes to uptake Fe^3+^ and endosomal transferring to Fe^2+^. Through Fenton reaction, Fe^2+^ promotes the production of ROS to aggravate lipid peroxidation. Some Fe^2+^ can also transfer into mitochondria via open channels such as mitochondrial permeability transition pore. In mitochondria, Fe^2+^ could also assist mitochondrial ROS production via Fenton reaction. The release of mitochondrial ROS further exaggerates the production of lipid peroxidation, which promotes ferroptosis in tubular cells and assists AKI progression.

In conclusion, our results suggest that CD44 plays an important role in the pathogenesis of AKI. Through the activation of MAPK and NF-κB p65, CD44 inhibits PGC-1α transcription, leading to mitochondrial ROS production and lipid accumulation, resulting in mitochondrial dysfunction and further triggering cell apoptosis. Additionally, CD44 facilitates the endosomal uptake of Fe^3+^, which transfers to Fe^2+^ in endolysosomes. Then, Fe^2+^ assists cell ferrotosis by promoting the production of mitochondrial ROS and lipid oxidation through the Fenton reaction, leading to ferroptosis and contributing AKI to some extent (Fig. 10).

## Discussion

Cell apoptosis is the most common pathological feature in AKI [2]. As the main cell type in renal parenchyma, TECs are vulnerable to undergo cellular senescence, dedifferentiation, apoptosis, and so on, due to their huge energy demand [12]. Mitochondria are the organelles to supply energy in eukaryotic cells. Hence, mitochondrial dysfunction plays a key role in renal tubular cell injury. However, the underlying mechanisms of TEC apoptosis and mitochondrial dysfunction still remain unclear.

Large quantities of studies have reported impaired mitochondrial biogenesis plays a vital role in TEC apoptosis, through ATP absence and generation of extensive ROS [14]. A recent study has found FAO dysfunction is also one of the important mechanisms for TEC apoptosis in AKI [6]. FAO deficiency not only contributes to insufficient ATP generation but also leads to lipid deposition, which collaboratively induces ROS production and tubular cell apoptosis. Notably, as shown in IRI mice, mitochondrial fragmentation and lipid accumulation were also found in our study (Fig. 3).

CD44 is a cell-surface glycoprotein with a wide range of functions. Numerous studies have found that the activation of the CD44 signaling pathway significantly affects cellular proliferation and differentiation. Moreover, CD44 also performs its function through the interaction with its ligands, such as hyaluronan (HA). It has been found that CD44 facilitates the intake of iron and Cu^2+^ due to the enrichment effect of hyaluronic acid on iron and Cu^2+^ [10, 11]. Through the binding of HA, CD44-positive inflammatory cells are recruited to regulate wound healing [8]. CD44 mediates the adhesion to HA, to further contribute to invasive motility [9, 20]. Previous studies have found CD44 plays a role in kidney inflammation and injury upon IRI. Our study consistently found that CD44 exacerbates tubular injury and kidney function decline (Figs. 2 and 5). The cell type highly expressing CD44 in AKI is controversial. In LPS-induced AKI animal models, CD44 mRNA level is upregulated and mainly located in the interstitium and sometimes in the glomeruli, while absent in TECs [21]. Another study believed that CD44 is highly expressed in peritubular endothelium and infiltrating cells in interstitium in the early IRI model, and later, CD44 is detected in leukocytes, endothelial, and TECs [22]. Other studies declared that CD44 was constantly expressed in TECs [3, 23]. In our study, we found that CD44 is highly expressed in TECs 1 day after IRI surgery and 3 days after cisplatin or glycerol injection (Fig. 1 and Supplementary Fig. S1).

Recent studies have found that CD44 can regulate glucose and lipid homeostasis. They found that silencing or ablation of CD44 can promote mitochondrial OXPHOS in breast and colorectal cancer cells [7]. CD44 also promotes the accumulation of lipid in the liver of high-fat diet (HFD)-fed obese mice, while another study observed that CD44 knockout exhibited increased lipid accumulation in adipose tissue of HFD-fed obese mice. In contrast, a study declared that CD44 promotes the accumulation of lipid droplets in preadipocytes in vitro [24]. The molecule mechanism of CD44 in metabolic regulation is still unclear and requires further investigation. In our study, we first found that CD44 aggravates mitochondrial dysfunction and FAO defects in renal tubular cells and kidney injury (Figs. 3, 6, and 7).

CD44 plays an important role in the intake of iron and Cu^2+^ through the interaction with HA, which is widely enriched in the kidney upon AKI [10, 11, 25], suggesting that CD44 may have effects on regulating ferroptosis and cuproptosis upon AKI. We first found that CD44 is prone to drive the intake of iron and Cu^2+^ in vitro and in vivo (Fig.9 and Supplementary Fig. S2). Furthermore, in vitro and vivo, we found CD44 could promote ferroptosis slightly (Fig. 9). Of note, we found CD44 knockout strongly inhibited cell apoptosis in both bioinformatic and biological analysis. Therefore, we think CD44 majorly functions in cell apoptosis and also slightly contributes to ferroptosis in the kidney system. Interestingly, CD44 did not affect the cuproptosis markers FDX1 and LIAS in AKI, although it promoted Cu^2+^ import (Supplementary Fig. S2). We thought that this lies in that CD44 has no effect on converting Cu^2+^ into Cu^+^, which leads to cuproptosis by disulfide bond formation. Thus, one would not expect cuproptosis to only result from increased Cu^2+^ accumulation.

Ferroptosis is a Fe^2+^-dependent cell death triggered by lipid peroxidation of PUFAs in membrane phospholipids, especially PE and PC. And then oxidized phospholipids are degraded or cleaved by phospholipase [26]. Moreover, FAO dysfunction would lead to the accumulation of glycerolipids. It implied that CD44 may have effects on regulating the lipid compositions of tubular cells. The results of our lipidomics sequencing implied that CD44 knockdown can restore the content of PE, PC, and cardiolipin while resulting in a decrease in triglyceride and diglyceride contents. In addition, CD44 knockdown also regulated the contents of other kinds of lipids, such as SM, Hex2cer, and so on, suggesting CD44 affects the whole processes of lipid metabolism (Fig. 8). Hence, the role of CD44 in lipid metabolism is worthy to be studied in the future.

Of note, ferroptosis and cuproptosis could affect other cell death models. Overload of iron and copper can trigger more oxidative stress and more mitochondrial ROS production, which further triggers mitochondrial dysfunction, lipid peroxidation and DNA double-strand breaks [27]. This would aggravate cell apoptosis [28]. Although CD44 promotes the import of iron and copper, we believe that the CD44-mediated overload of iron and copper mainly promotes cellular apoptosis via ROS-induced mitochondrial dysfunction and lipid peroxidation.

PGC-1α is the major regulator of mitochondrial biogenesis [29]. As a co-transcriptional regulation factor, PGC-1α triggers mitochondrial biogenesis by activating different transcription factors, such as NRF-1 and NRF-2, which promote the expression of proteins involved in TCA cycle and OXPHOS systems. Moreover, PGC-1α can also regulate glucose utilization and FAO via interacting with other transcription factors, such as PPARα, estrogen-related receptors (ERRs), and thyroid hormone. When PGC-1α docks on PPARα, it enhances the transcriptional activities of PPARα, contributing to the expression of FAO-related proteins, such as CPT1, CPT2, and so on [14]. Reports have shown the mRNA and protein levels of PGC-1α are both downregulated in TECs in AKI mice, contributing to mitochondrial dysfunction and FAO deficiency. However, the molecular mechanism requires further investigation.

NF-κB p65, a transcription factor, is associated with tubular cell apoptosis and AKI progression [30, 31]. Apart from the canonical role of NF-κB as a transcriptional activator, it is also able to suppress the transcription activities of promoters via the recruitment of chromatin-modifying proteins. For example, the class I histone deacetylases (HDACs) can interact with NF-κB p65 and form a protein complex, which transcriptionally silences target genes [16, 32, 33]. Recent studies have found that NF-κB p65 could downregulate PGC-1α expression [16, 34]. It was found that hypoxia induces NF-κB p65 binding to PGC-1α promoter region in cardiac myocytes, contributing to the transcription silence of PGC-1α [16]. However, it is unknown whether p65 plays a similar role in renal tubular cells. In our study, we found that H/R promotes the phosphorylation and nuclear translocation of NF-κB p65, which then docks on the PGC-1α promoter to suppress PGC-1α promoter activity (Fig. 8).

The MAPK signaling pathway, which is activated by signal transduction from cytoplasm receptors and then transfers signals into the nucleus, plays an important role in the development of AKI [35, 36]. The members of MAPK include ERK1/2, JNK, and p38. The transcription sequencing profile in our studies implied that the MAPK signaling pathway is deactivated in CD44 knockout mice, suggesting CD44 may trigger AKI through MAPK signaling. Indeed, we found ectopic CD44 significantly activated p38 and ERK1/2, but not JNK. Recent studies found that p-ERK1/2 and p-p38 are able to phosphorylate and activate NF-κB p65 [37, 38]. Consistently, our study also found that CD44 activated ERK1/2 and p38, which then phosphorylate NF-κB p65 and triggered the downregulation of PGC-1α (Fig. 8).

Taken together, our study showed that the aberrant expression of CD44 plays a significant role in mitochondrial dysfunction and FAO defects, which further contributes to tubular cell apoptosis in IRI. This effect is mediated by p-ERK1/2- and p-p38-induced activation of NF-κB p65, which subsequently results in the transcriptional silence of PGC-1α. Although more studies are required, our study provides a new mechanism and a prospective therapeutic strategy for AKI.

## Materials and methods

### Animal model

The animal experiments were approved by the Ethics Committee on Use and Care of Animals of Southern Medical University, Guangzhou, China. Eight-week-old male C57BL/6N mice were purchased from Southern Medical University Animal Center (Guangzhou, China), and maintained under a standard environment. CD44 conventional knockout mice in C57BL/6N background were purchased from Cyagen Biosciences (stock no. KOCMP-12505-Cd44-B6N, Cyagen Biosciences, Guangzhou, China). Mice were randomized into different groups using a random number table. Five mice were included in each group to meet the minimum sample size requirement to perform an independent sample *t*-test and one-way ANOVA analysis. Mice were performed by IRI surgery, intramuscular injection of glycerol, or intraperitoneal injection of cisplatin, as described in previous studies [2, 39].

For IRI model, bilateral renal pedicles were clipped for 30 min by microaneurysm clamps at 37.8 °C during the ischemia period. Mice were euthanized 24 h after IRI surgery. Some mice were injected with pcDNA3 or p-HA-CD44 overexpression plasmid via the tail vein 3 days before IRI surgery. For rhabdomyolysis-induced AKI, 50% glycerol at the dose of 7.5 ml/kg is administered by a single intramuscular injection. Mice were euthanized 3 days after glycerol injection. For cisplatin-induced AKI, mice were intraperitoneally injected with cisplatin at the dose of 20 mg/kg. Mice were euthanized 3 days after cisplatin injection. Serum and kidney tissues were collected for the following analyses.

### Determination of Scr and BUN

The levels of Scr and BUN were measured with an AU480 Automatic biochemical analyzer (Beckman Coulter, Brea, CA) and were expressed as milligrams per 100 ml.

### Cell culture and treatment

HKC-8 cells were given by Dr. L. Racusen (Johns Hopkins University, Baltimore, MD, USA) and were performed as previously described [2]. The empty vector (pcDNA3), CD44 overexpression plasmid (pHA-CD44), p65 overexpression plasmid (pFlag-p65), control-shRNA or CD44-shRNA (5′-ATTTGAATATAACCTGCCG-3′) was transfected by Lipofectamine 2000 reagent (Invitrogen, Grand Island, NY, USA). Cells were incubated with erastin (HY-15763; MCE) (5 μM), PD98059 (HY-12028; MCE) (10 μM), SB203580 (HY-10256; MCE) (10 μM) or PDTC (HY-18738; MCE) (20 μM). The H/R was performed with routine protocol. HKC-8 cells were incubated in basal culture medium in a 1% O_2_ environment for 24 h and then were reoxygenated in normal O_2_ for 6 h.

### Western blot analysis

Protein expression was analyzed by western blot analysis. The primary antibodies were as follows: anti-BAX (SC-7480; Santa Cruz Biotechnology), anti-BCL2 (T40056; Abmart), anti-Caspase 3 (T40044; Abmart), anti-CD44 (A00052; Boster), anti-CPT1a (ab128568; Abcam), anti-ERK1/2 (4695; Cell Signaling Technology), anti-Flag-tag (M185-3S; MBL), anti-GAPDH (RM2001; Ray Antibody Biotech), anti-JNK (9252; Cell Signaling Technology), anti-NGAL (ab63929; Abcam), anti-p-ERK1/2 (4370; Cell Signaling Technology), anti-PGC-1α (A20995; Abclonal), anti-p-JNK (4668; Cell Signaling Technology), anti-PPARα (A24853; Abclonal), anti-p-p38 (AP0526; Abclonal), anti-p-p65 (82335-1-RR; Proteintech), anti-p38 (A14401; Abclonal), anti-p65 (8242; Cell Signaling Technology), anti-TOMM20 (ab186735;Abcam), anti-α-tubulin (RM2007; Ray Antibody Biotech, Beijing, China), anti-β-actin (RM2001; Beijing Ray Antibody Biotech).

### Histology and immunohistochemical staining

Paraffin kidney sections (3 μm) were stained with PAS reagent by standard protocol. Immunohistochemical staining was performed with routine protocol [2]. Antibodies used were as follows: anti-KIM-1 (BA3537; Boster), anti-NGAL (ab63929; Abcam), anti-p-ERK1/2 (4370; Cell Signaling Technology), anti-p-p38 (AP0526; Abclonal).

### Immunofluorescence staining

Immunofluorescence staining was performed with routine protocol [2]. Frozen kidney sections (3 μm) and cells cultured on coverslips were fixed with 4% paraformaldehyde. All primary antibodies used were as follows: rabbit anti-CD44 (A00052; Boster), rabbit anti-Cleaved caspase 3 (25128-1-AP; Proteintech), rabbit anti-Perilipin 2 (A20843; Abclonal), rabbit anti-PPARα (A24853; Abclonal), rabbit anti-p-p38 (AP0526; Abclonal), rabbit anti-p-p65 (AP0475; Abclonal), rabbit anti-p65 (8242; Cell Signaling Technology), rabbit anti-TOMM20 (ab186735; Abcam), mouse anti-CD44 (60224-1-Ig; Proteintech), mouse anti-CPT1a (ab128568; Abcam), mouse anti-PGC-1α (66368-1-Ig; Proteintech), mouse anti-TOMM20 (66777-1-Ig; Proteintech), rat anti-CD44 (AB119348; Abcam), anti-LTL (FL-1321; VECTOR Laboratories), anti-PNA (FL-1071; VECTOR Laboratories), anti-DBA (FL1031; VECTOR Laboratories).

### Transmission electron microscopy (TEM)

Kidney tissues were fixed in 1.25% glutaraldehyde in phosphate buffer. Ultrathin sections (60 nm) were prepared by a routine procedure. The mitochondria and lipid droplets were observed under an electron microscope (JEOL JEM-1010, Tokyo, Japan).

### Nile red staining

Cells cultured on coverslips were stained with Nile red according to the manufacturer’s instructions. Cells were fixed with 4% paraformaldehyde for 10 min at room temperature and then were stained by Nile red (1 μg/ml) dissolved in DAPI after washed by PBS.

### Oil Red O staining

1.5 g of Oil Red O (1320-06-5; Sigma) was dissolved in 100 ml of isopropanol and mix for 2 h at room temperature. Add one part of distilled water into 1.5 parts Oil Red O solution. Mixing the solution for 30 min at room temperature. And then filter the solution 3 times to remove precipitates. Frozen kidney sections (10 μm) were fixed with 4% paraformaldehyde for 30 min. Incubating frozen kidney sections with Oil Red O solution for 15 min [40].

### TUNEL assay

Frozen kidney sections (3 μm) or cells cultured on coverslips were assessed by TUNEL assay (G3250; Promega), according to the manufacturer’s instruction.

### FerroOrange staining

Cells were stained by FerroOrange (F374; dojindo) (1 μM) for 30 min, according to the manufacturer’s instruction, and were taken the images immediately.

### MitoSox staining

Frozen kidney sections (3 μm) or cells cultured on coverslips were assessed by MitoSOX Red (40778ES50; Yeasen), according to the manufacturer’s instruction.

### MDA assay

Lipid peroxidation assays were performed using a lipid peroxidation (MDA) assay kit (S0131; Beyotime) according to the manufacturer’s instructions.

### Iron and copper assay

Total iron (Fe^2+^, Fe^3+^) were measured using an iron assay kit (G4301; Servicebio) and Cu^2+^ was measured using a copper assay kit (BC5565; Solarbio), according to manufacturer’s instructions.

### Reverse transcriptase (RT) and real-time PCR

The sequences of the primer pairs used in quantitative real-time PCR are described in Supplementary Table 1.

### Chromatin immunoprecipitation (ChIP)

ChIP was performed using the Simple ChIP Plus (Magnetic Bead) Kit (9005, Cell Signaling Technology), according to the manufacturer’s instructions. HKC-8 cells were transfected with p65 overexpression plasmid (pFlag-p65) for 24 h. The antibody against p65 (A00284-1; Boster), normal rabbit IgG, and H3 was added and incubated overnight at 4 °C, followed by incubation with ChIP-Grade Protein G Magnetic Beads for 2 h. After washing out the precipitate, purified DNA was amplified as a template by PCR. The sequences of human PGC-1α primers were as follows: forward 5′-TGACAGCCCAGCCTACTTT-3′ and reverse 5′-TTTTCAACTCCAATCCACA-3.

### Statistical analyses

Statistical analysis was performed by a researcher who was blinded. All data were expressed as mean ± SEM. Statistical analysis was carried out by SPSS 25.0 (SPSS Inc., Chicago, IL, USA). Independent sample *t*-test was used to compare the mean of the two groups. Comparison between groups was made via one-way ANOVA analysis of variance followed by the least significant difference when the variance between groups was homogeneous, or the Dunnett T3 test when the variance between groups was not homogeneous. Bivariate correlation analysis was performed using Pearson correlation analysis. *P* value < 0.05 was considered as significant.

## Supplementary information

Supplementary table 1

Supplementary figure legends

Supplementary figure s1

Supplementary figure s2

Original Data File

## Data Availability

RNA Sequencing data produced in this study has been uploaded to the NCBI GEO database (accession number: GSE252060). Lipidomics Sequencing data in this study has been upload in MetaboLights database (accession number: MTBLS10954). The data used to support the findings of this study are available from the corresponding author upon request.

## References

[1] Singbartl K, Kellum JA. AKI in the ICU: definition, epidemiology, risk stratification, and outcomes. Kidney Int. 2012;81:819–25. 21975865 10.1038/ki.2011.339

[2] Miao J, Huang J, Liang Y, Zhang Y, Li J, Meng P, et al. Sirtuin 6 is a key contributor to gender differences in acute kidney injury. Cell Death Discov. 2023;9:134. 37185276 10.1038/s41420-023-01432-y PMC10130034

[3] Chen TH, Liu CT, Cheng CY, Sue YM, Huang NJ, Chen CH. Oligosaccharides ameliorate acute kidney injury by alleviating cluster of differentiation 44-mediated immune responses in renal tubular cells. Nutrients. 2022;14:760. 35215410 10.3390/nu14040760 PMC8877265

[4] Livingston MJ, Shu S, Fan Y, Li Z, Jiao Q, Yin XM, et al. Tubular cells produce FGF2 via autophagy after acute kidney injury leading to fibroblast activation and renal fibrosis. Autophagy. 2023;19:256–77. 35491858 10.1080/15548627.2022.2072054 PMC9809951

[5] Chung KW, Lee EK, Lee MK, Oh GT, Yu BP, Chung HY. Impairment of PPARα and the fatty acid oxidation pathway aggravates renal fibrosis during aging. J Am Soc Nephrol. 2018;29:1223–37. 29440279 10.1681/ASN.2017070802 PMC5875952

[6] Li M, Li CM, Ye ZC, Huang J, Li Y, Lai W, et al. Sirt3 modulates fatty acid oxidation and attenuates cisplatin-induced AKI in mice. J Cell Mol Med. 2020;24:5109–21. 32281286 10.1111/jcmm.15148 PMC7205836

[7] Weng X, Maxwell-Warburton S, Hasib A, Ma L, Kang L. The membrane receptor CD44: novel insights into metabolism. Trends Endocrinol Metab. 2022;33:318–32. 35249813 10.1016/j.tem.2022.02.002

[8] Aya KL, Stern R. Hyaluronan in wound healing: rediscovering a major player. Wound Repair Regen. 2014;22:579–93. 25039417 10.1111/wrr.12214

[9] Turley EA. Hyaluronan and cell locomotion. Cancer Metastasis Rev. 1992;11:21–30. 1380898 10.1007/BF00047600

[10] Solier S, Müller S, Cañeque T, Versini A, Mansart A, Sindikubwabo F, et al. A druggable copper-signalling pathway that drives inflammation. Nature. 2023;617:386–94. 37100912 10.1038/s41586-023-06017-4 PMC10131557

[11] Müller S, Sindikubwabo F, Cañeque T, Lafon A, Versini A, Lombard B, et al. CD44 regulates epigenetic plasticity by mediating iron endocytosis. Nat Chem. 2020;12:929–38. 32747755 10.1038/s41557-020-0513-5 PMC7612580

[12] Chen S, Zhang M, Li J, Huang J, Zhou S, Hou X, et al. β-catenin-controlled tubular cell-derived exosomes play a key role in fibroblast activation via the OPN-CD44 axis. J Extracell Vesicles. 2022;11:e12203. 35312232 10.1002/jev2.12203 PMC8936047

[13] Hamatani H, Eng DG, Hiromura K, Pippin JW, Shankland SJ. CD44 impacts glomerular parietal epithelial cell changes in the aged mouse kidney. Physiol Rep. 2020;8:e14487. 32597007 10.14814/phy2.14487 PMC7322268

[14] Huang J, Liang Y, Zhou L. Natural products for kidney disease treatment: focus on targeting mitochondrial dysfunction. Front Pharmacol. 2023;14:1142001. 37007023 10.3389/fphar.2023.1142001 PMC10050361

[15] Markó L, Vigolo E, Hinze C, Park JK, Roël G, Balogh A, et al. Tubular epithelial NF-κB activity regulates ischemic AKI. J Am Soc Nephrol. 2016;27:2658–69. 26823548 10.1681/ASN.2015070748 PMC5004652

[16] Rabinovich-Nikitin I, Blant A, Dhingra R, Kirshenbaum LA, Czubryt MP. NF-κB p65 attenuates cardiomyocyte PGC-1α expression in hypoxia. Cells. 2022;11:2193. 35883637 10.3390/cells11142193 PMC9322255

[17] Li H, Dixon EE, Wu H, Humphreys BD. Comprehensive single-cell transcriptional profiling defines shared and unique epithelial injury responses during kidney fibrosis. Cell Metab. 2022;34:1977–98.e1979. 36265491 10.1016/j.cmet.2022.09.026 PMC9742301

[18] Miao J, Huang J, Luo C, Ye H, Ling X, Wu Q, et al. Klotho retards renal fibrosis through targeting mitochondrial dysfunction and cellular senescence in renal tubular cells. Physiol Rep. 2021;9:e14696. 33463897 10.14814/phy2.14696 PMC7814487

[19] Jia D, Zhang J, Nie J, Andersen JP, Rendon S, Zheng Y, et al. Cardiolipin remodeling by ALCAT1 links hypoxia to coronary artery disease by promoting mitochondrial dysfunction. Mol Ther. 2021;29:3498–511. 34111561 10.1016/j.ymthe.2021.06.007 PMC8636157

[20] Kim Y, Kumar S. CD44-mediated adhesion to hyaluronic acid contributes to mechanosensing and invasive motility. Mol Cancer Res. 2014;12:1416–29. 24962319 10.1158/1541-7786.MCR-13-0629 PMC4201971

[21] Rampanelli E, Dessing MC, Claessen N, Teske GJ, Joosten SP, Pals ST, et al. CD44-deficiency attenuates the immunologic responses to LPS and delays the onset of endotoxic shock-induced renal inflammation and dysfunction. PLoS ONE. 2013;8:e84479. 24376813 10.1371/journal.pone.0084479 PMC3871539

[22] Rouschop KM, Roelofs JJ, Claessen N, da Costa Martins P, Zwaginga JJ, Pals ST, et al. Protection against renal ischemia reperfusion injury by CD44 disruption. J Am Soc Nephrol. 2005;16:2034–43. 15901765 10.1681/ASN.2005010054

[23] Kocak B, Orug T, Turhan N, Ozcay N, Gonenc F. CD44 expression in renal ischemia-reperfusion injury in rats. Int Urol Nephrol. 2009;41:791–4. 19283506 10.1007/s11255-009-9542-0

[24] Wang M, Guo Y, Zhou Y, Yuan W, Li H, Xiong S, et al. Secreted-Osteopontin Contributes to Brown Adipogenesis In Vitro via a CD44-Dependent Pathway. Horm Metab Res = Hormon Stoffwechselforsch = Hormon Metab. 2019;51:741–8. 10.1055/a-0926-3991 31295749

[25] Zhang Y, Zhao H, Zhang J. Hyaluronidase inhibitor sHA2.75 alleviates ischemia-reperfusion-induced acute kidney injury. Cell Cycle (Georgetown, TX). 2024;23:248–61. 10.1080/15384101.2024.2309019 PMC11057651 38526145

[26] Kim JW, Lee JY, Oh M, Lee EW. An integrated view of lipid metabolism in ferroptosis revisited via lipidomic analysis. Exp Mol Med. 2023;55:1620–31. 37612411 10.1038/s12276-023-01077-y PMC10474074

[27] Zhao Y, Yang M, Liang X. The role of mitochondria in iron overload-induced damage. J Transl Med. 2024;22:1057. 39587666 10.1186/s12967-024-05740-4 PMC11587765

[28] Li Y, Du Y, Zhou Y, Chen Q, Luo Z, Ren Y, et al. Iron and copper: critical executioners of ferroptosis, cuproptosis and other forms of cell death. Cell commun Signal. 2023;21:327. 37974196 10.1186/s12964-023-01267-1 PMC10652626

[29] Jornayvaz FR, Shulman GI. Regulation of mitochondrial biogenesis. Essays Biochem. 2010;47:69–84. 20533901 10.1042/bse0470069 PMC3883043

[30] Ren Q, Guo F, Tao S, Huang R, Ma L, Fu P. Flavonoid fisetin alleviates kidney inflammation and apoptosis via inhibiting Src-mediated NF-κB p65 and MAPK signaling pathways in septic AKI mice. Biomed Pharmacother = Biomed Pharmacother. 2020;122:109772. 31918290 10.1016/j.biopha.2019.109772

[31] Ming S, Tian J, Ma K, Pei C, Li L, Wang Z, et al. Oxalate-induced apoptosis through ERS-ROS-NF-κB signalling pathway in renal tubular epithelial cell. Mol Med (Cambridge, MA). 2022;28:88. 10.1186/s10020-022-00494-5 PMC9347104 35922749

[32] Ashburner BP, Westerheide SD, Baldwin AS Jr. The p65 (RelA) subunit of NF-kappaB interacts with the histone deacetylase (HDAC) corepressors HDAC1 and HDAC2 to negatively regulate gene expression. Mol Cell Biol. 2001;21:7065–77. 11564889 10.1128/MCB.21.20.7065-7077.2001 PMC99882

[33] Baetz D, Regula KM, Ens K, Shaw J, Kothari S, Yurkova N, et al. Nuclear factor-kappaB-mediated cell survival involves transcriptional silencing of the mitochondrial death gene BNIP3 in ventricular myocytes. Circulation. 2005;112:3777–85. 16344406 10.1161/CIRCULATIONAHA.105.573899

[34] Alvarez-Guardia D, Palomer X, Coll T, Davidson MM, Chan TO, Feldman AM, et al. The p65 subunit of NF-kappaB binds to PGC-1alpha, linking inflammation and metabolic disturbances in cardiac cells. Cardiovasc Res. 2010;87:449–58. 20211864 10.1093/cvr/cvq080

[35] Moon H, Ro SW. MAPK/ERK signaling pathway in hepatocellular carcinoma. Cancers. 2021;13:3026. 34204242 10.3390/cancers13123026 PMC8234271

[36] Kim DU, Kweon B, Oh JY, Seo CS, Kim DG, Kim HY, et al. Ojeoksan ameliorates cisplatin-induced acute kidney injury in mice by downregulating MAPK and NF-κB pathways. Int J Mol Sci. 2022;23:12254. 36293111 10.3390/ijms232012254 PMC9603434

[37] Liu C, Hu F, Jiao G, Guo Y, Zhou P, Zhang Y, et al. Dental pulp stem cell-derived exosomes suppress M1 macrophage polarization through the ROS-MAPK-NFκB P65 signaling pathway after spinal cord injury. J Nanobiotechnol. 2022;20:65. 10.1186/s12951-022-01273-4 PMC8811988 35109874

[38] Zhang D, Shi R, Xiang W, Kang X, Tang B, Li C, et al. The Agpat4/LPA axis in colorectal cancer cells regulates antitumor responses via p38/p65 signaling in macrophages. Signal Transduct Target Ther. 2020;5:24. 32296017 10.1038/s41392-020-0117-y PMC7099097

[39] Shen K, Miao J, Gao Q, Ling X, Liang Y, Zhou Q, et al. Annexin A2 plays a key role in protecting against cisplatin-induced AKI through β-catenin/TFEB pathway. Cell Death Discov. 2022;8:430. 36307397 10.1038/s41420-022-01224-w PMC9616836

[40] Mehlem A, Hagberg CE, Muhl L, Eriksson U, Falkevall A. Imaging of neutral lipids by oil red O for analyzing the metabolic status in health and disease. Nat Protoc. 2013;8:1149–54. 23702831 10.1038/nprot.2013.055

