# Supplementary material for: Tubular CD44 plays a key role in aggravating AKI through NF-κB p65-mediated mitochondrial dysfunction
Source: Cell Death Dis. 2025 Feb 20;16(1):119. doi: 10.1038/s41419-025-07438-x (PMC11842857; doi:10.1038/s41419-025-07438-x)
Supplement: Supplementary file 1 — Supplementary table 1 [file 41419_2025_7438_MOESM1_ESM.docx]

***Supplementary table 1. Nucleotide sequence of the primer used for qRT-PCR***

| Primer Sequence (5' to 3')  Gene Forward Reverse |
| --- |
| Mouse:  β-actin 5'-CAGCTGAGAGGGAAATCGTG -3' 5'-CGTTGCCAATAGTGATGACC -3'  ACOX1 5'-CTTGGATGGTAGTCCGGAGA -3' 5'-TGGCTTCGAGTGAGGAAGTT-3'  ACSL4 5'-CGGGAGATCCTGAGTGAAGAAA-3' 5'-TGGCAATGGTGTTCTTTGGTTT -3'  CPT1 5'-GGTCTTCTCGGGTCGAAAGC-3' 5'-TCCTCCCACCAGTCACTCAC-3'  CPT2 5'-CAATGAGGAAACCCTGAGGA-3' 5'- GATCCTTCATCGGGAAGTCA -3'  FDX1 5'-GCGGAGCAGCTCAGAAGAT-3' 5'-AGCCAACGTTCCCTCACAC-3'  GPX4 5'-ACGAATTCTCAGCCAAGGACAT-3' 5'-ATGCAGATCGACTAGCTGAGTG -3'  LIAS 5'-CAGTCGAGAAGGTGGCTCTG-3' 5'-GGAAACAACATCAGGCTGGA-3'  PGC-1α 5'- AGTCCCATACACAACCGCAG-3' 5'-CCCTTGGGGTCATTTGGTGA-3'  PPARα 5'-TGCAAACTTGGACTTGAACG-3' 5'-GATCAGCATCCCGTCTTTGT-3'    Human  β-actin 5'-CTCACCATGGATGATGATATCGC-3' 5'-AGGAATCCTTCTGACCCATGC-3'  ACSL4 5'-AATGCAGCCAAATGGAAAA-3' 5'-GCCCTGGTCTCACAGAAGA-3'  CD44 5'-TCCACCATTGAGAAGAGCACC-3' 5'-CTGAGCTGTTGCATGGCTTTT-3'  FDX1 5'-AACCCTGGCTTGTTCAACCT-3' 5'-CCCAACCGTGATCTGTCTGT-3'  GPX4 5'-CTCCATGCACGAGTTTTCC-3' 5'-AGGTCGACGAGCTGAGTGT-3'  LIAS 5'-GGAAGCTCGATGTCCCAATA-3' 5'-TAATCCAGACCCCATTCTGC-3'  PGC-1α 5'-TGTGCAACTCTCTGGAACTG-3' 5'-TGAGGACTTGCTGAGTGGTG-3' |
