# Supplementary material for: Tubular CD44 plays a key role in aggravating AKI through NF-κB p65-mediated mitochondrial dysfunction
Source: Cell Death Dis. 2025 Feb 20;16(1):119. doi: 10.1038/s41419-025-07438-x (PMC11842857; doi:10.1038/s41419-025-07438-x)
Supplement: Supplementary file 2 — Supplementary figure legends [file 41419_2025_7438_MOESM2_ESM.docx]

**Supplementary figure legends**

**Supplementary figure S1.**

**(A-B)** Representative western blot of CD44 (A) and graphical representations (B) protein expression levels are shown. ****P* < 0.001 versus control group (n=5). **(C)** Representative micrographs show the expression of CD44 in control and cisplatin groups, as indicated. Frozen kidney sections were stained with an antibody against CD44. Arrows indicate positive staining. Scale bar, 50 μm. **(D-E)** Representative western blot of CD44 (D) and graphical representations (E) protein expression levels are shown. ****P* < 0.001 versus control group (n=5). **(F)** Representative micrographs show the expression of CD44 in control and rhabdomyolysis groups, as indicated. Frozen kidney sections were stained with an antibody against CD44. Arrows indicate positive staining. Scale bar, 50 μm. (**G**) Pearson correlation between the relative fluorescence intensity between CD44 and TOMM20. (**H**) Co-localization of CD44 and cleaved caspase 3 in sham group. Frozen renal sections were subjected to immunostaining of CD44 (red) and Cleaved caspase 3 (green). Scale bar, 50 μm. (**I**) Pearson correlation between the relative fluorescence intensity between CD44 and cleaved caspase 3. (**J**) Identified reports of CD44 knockout mice. For Cre primer, the wild-type mice have no band, while CD44 knockout mice have one band with 534bp. For Loxp primer, wide-type mice have one band with 418bp, while CD44 knockout mice have one band with 534bp. (**K**) Representative heatmap gene expression of RNA sequencing analysis show that CD44 is involved with apoptosis, mitochondria and FAO. (**L-M**) HKC-8 cells were transfected with pcDNA3 or p-HA-CD44 for 24 hours. Representative western blot (L) and graphical representations of p-ERK1/2/ERK1/2, p-p38/p38, p-JNK/JNK and p-p65 protein expression levels are shown. n.s., no significant differences, **P* < 0.05, ***P* < 0.01, ****P* < 0.001 versus pcDNA3 groups (n=3). (**N**) Pearson correlation between the relative fluorescence intensity between CD44 and p65. (**O**) Representative micrographs show the expression of PGC-1α, TOMM20 and PPARα, mitochondrial ROS via MitoSox staining in different groups, as indicated. Arrows indicate positive staining. Frozen kidney sections were subjected to MitoSox staining and stained with antibody against PGC-1α, TOMM20 and PPARα. Scale bar, 50 μm. (**P**) Graphic presentation shows the relative mRNA levels of CD44 in different groups as indicated. *P < 0.05 versus Crtl-shR group; ^#^P < 0.05 versus H/R with Crtl-shR group (n = 3).

**Supplementary figure S2.**

(**A**) Pearson correlation between the relative fluorescence intensity between CD44 and cleaved caspase 3 in HKC-8 cells. (**B-C**) Representative western blot (B) and graphical representations of (C) TOMM20 protein expression levels are shown. ***P* < 0.01 versus pcDNA3 group; ^#^*P* < 0.05 versus H/R with pcDNA3 group (n = 3). (**D**) Quantitative result showing Cu^2+^ content among different groups. **P* < 0.05 versus Ctrl-shR group; ^#^*P* < 0.05 versus H/R with ctrl-shR (n = 3). (**E-F**) Quantitative result of QPCR showing relative (E) FDX1 and (F) LIAS mRNA level among different groups. ****P* < 0.001 versus Ctrl-shR group; n.s., no significant differences versus H/R with ctrl-shR (n = 3). (**G**) Quantitative result showing Cu^2+^ content among different groups. **P* < 0.05 versus pcDNA3 group; ^#^*P* < 0.05 versus H/R with pcDNA3 (n = 3). (**H-I**) Quantitative result of QPCR showing relative (H) FDX1 and (I) LIAS mRNA level among different groups. ***P* < 0.01 versus pcDNA3 group; n.s., no significant differences versus H/R with pcDNA3 (n = 3). (**J**) Quantitative result showing Cu^2+^ content in kidney tissue among different groups. ****P* < 0.001 versus wild-type mice upon sham group; ^###^*P* < 0.001 versus wild-type mice upon IRI group (n = 5). (**K-L**) Quantitative result of QPCR showing relative (K) FDX1 and (L) LIAS mRNA level among different groups. ****P* < 0.001 versus wild-type mice upon sham group; n.s., no significant differences versus wild-type mice upon IRI group (n = 5). (**M**) Quantitative result showing Cu^2+^ content in kidney tissue among different groups. ****P* < 0.001 versus sham group; ^##^*P* < 0.01 versus IRI group injected with pcDNA3 (n = 5). (**N-O**) Quantitative result of QPCR showing relative (N) FDX1 and (O) LIAS mRNA level among different groups. ****P* < 0.001 versus w sham group; n.s., no significant differences versus IRI group injected with pcDNA3 (n = 5).
