# Supplementary figures and images for: Tubular CD44 plays a key role in aggravating AKI through NF-κB p65-mediated mitochondrial dysfunction
Source: Cell Death Dis. 2025 Feb 20;16(1):119. doi: 10.1038/s41419-025-07438-x (PMC11842857; doi:10.1038/s41419-025-07438-x)

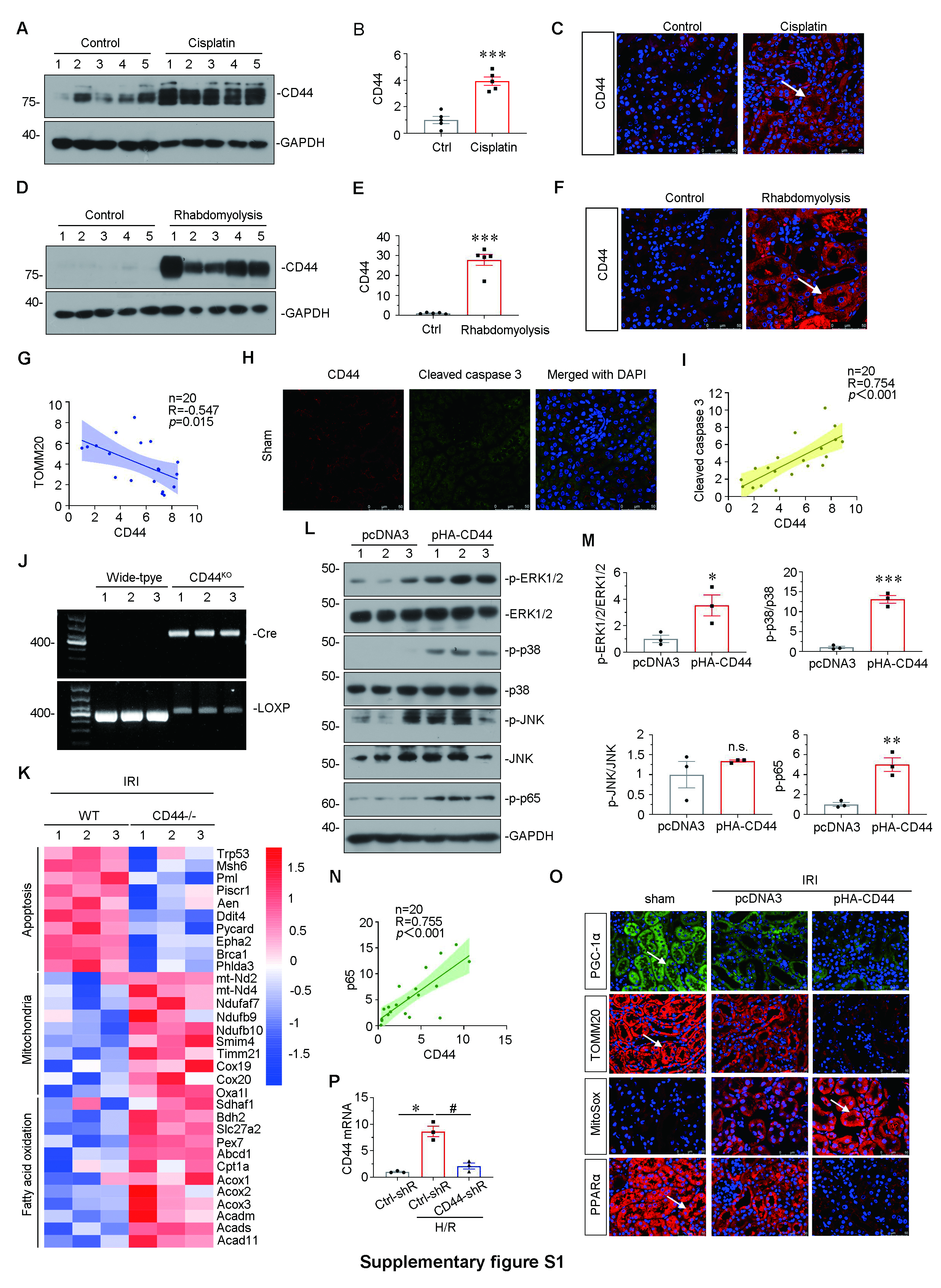

Supplement: Supplementary file 3 — Supplementary figure s1 [file 41419_2025_7438_MOESM3_ESM.jpg]

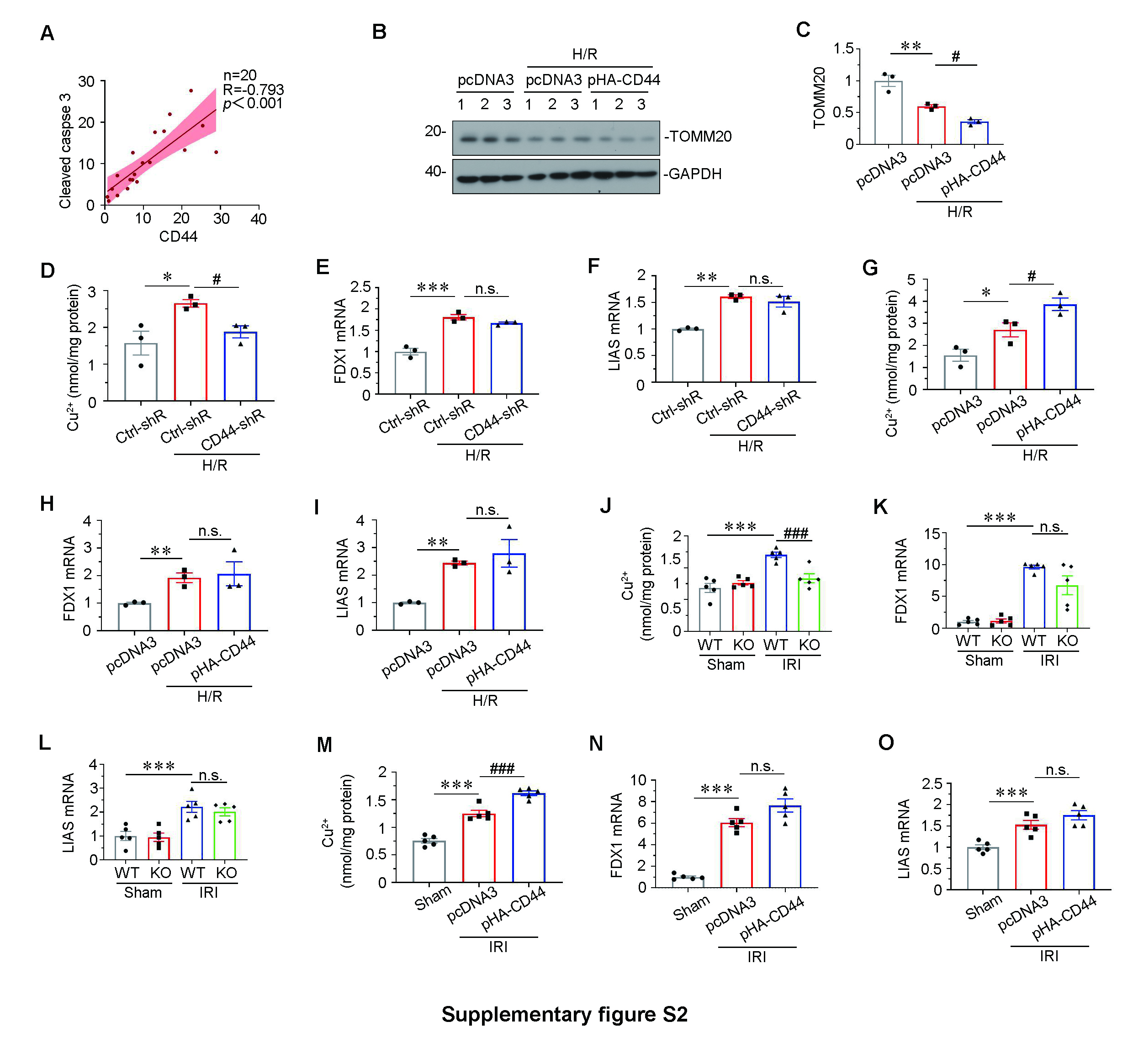

Supplement: Supplementary file 4 — Supplementary figure s2 [file 41419_2025_7438_MOESM4_ESM.jpg]
