# Supplementary material for: Tubular CD44 plays a key role in aggravating AKI through NF-κB p65-mediated mitochondrial dysfunction
Source: Cell Death Dis. 2025 Feb 20;16(1):119. doi: 10.1038/s41419-025-07438-x (PMC11842857; doi:10.1038/s41419-025-07438-x)

Full unedited gels for Figure 1D

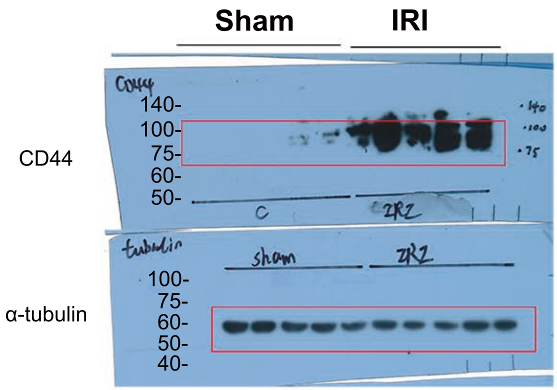

Full unedited gels for Figure 2D

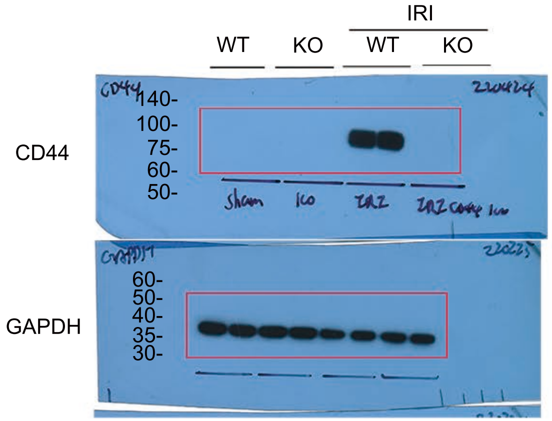

Full unedited gels for Figure 2I

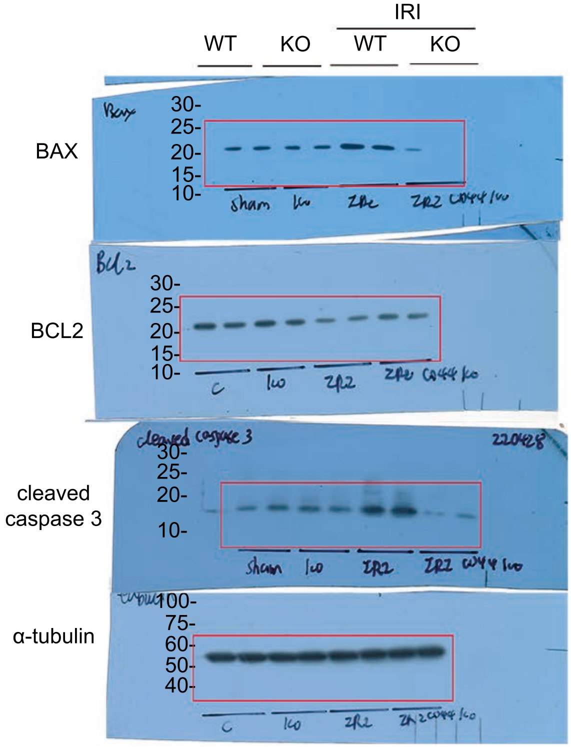

Full unedited gels for Figure 2N

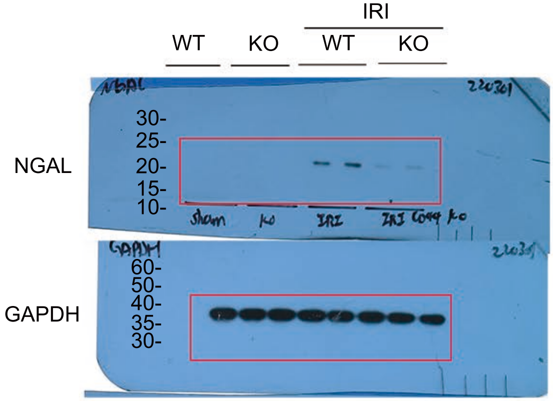

Full unedited gels for Figure 4E

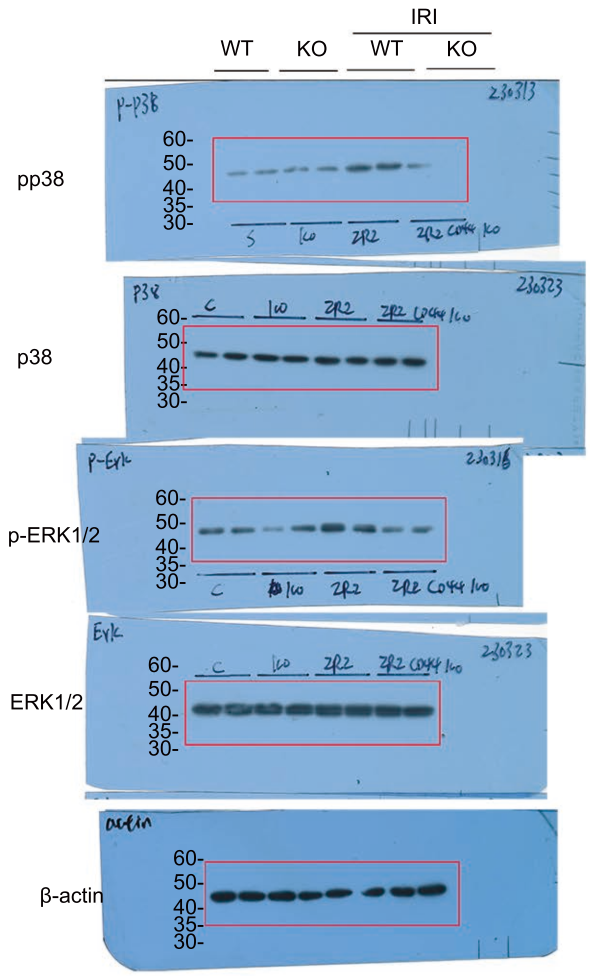

Full unedited gels for Figure 4H

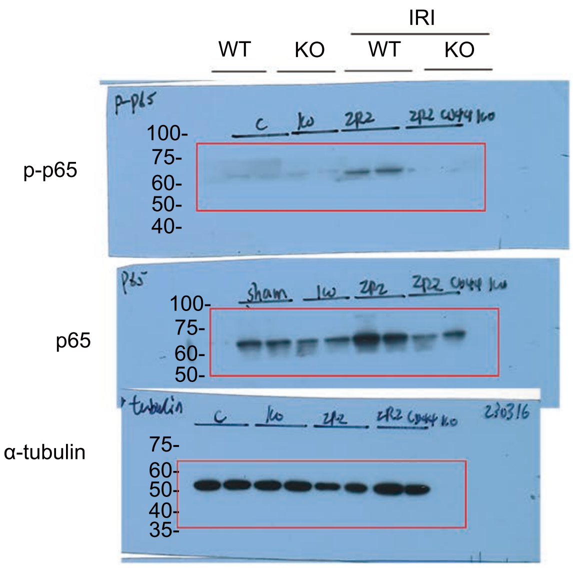

Full unedited gels for Figure 3D

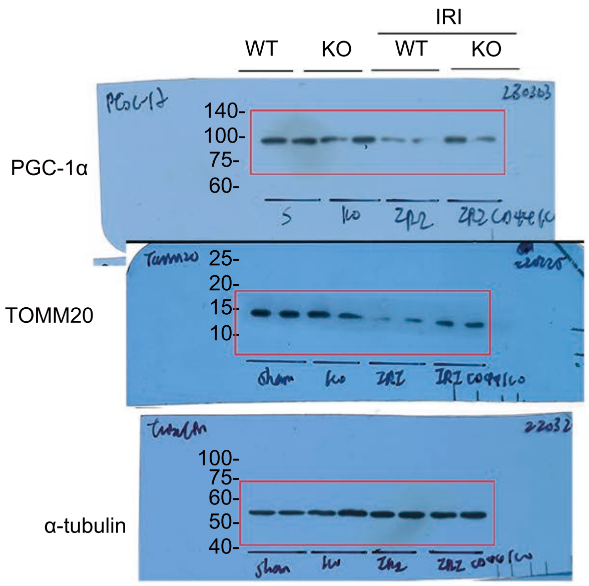

Full unedited gels for Figure 3J

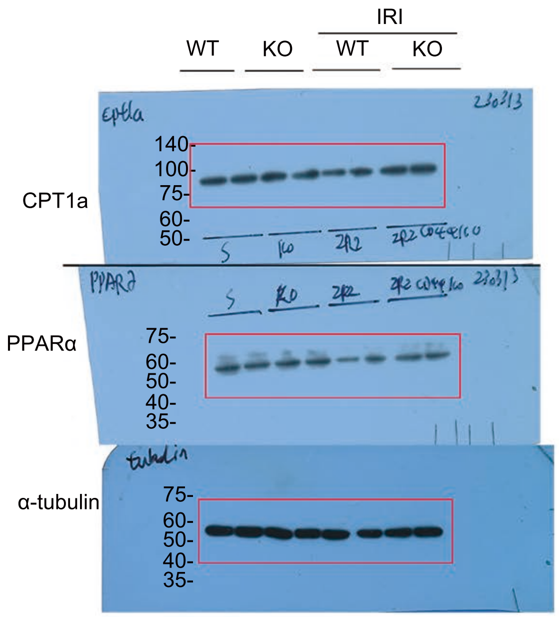

Full unedited gels for Figure 5D

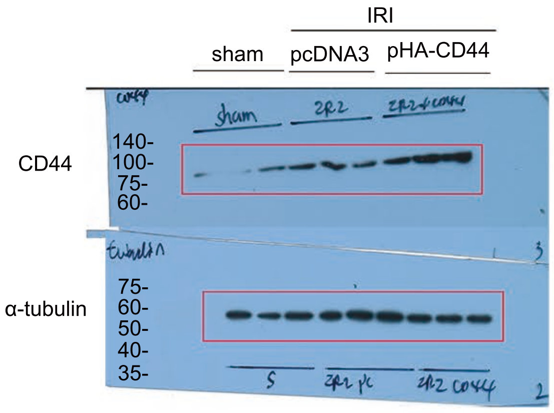

Full unedited gels for Figure 5G

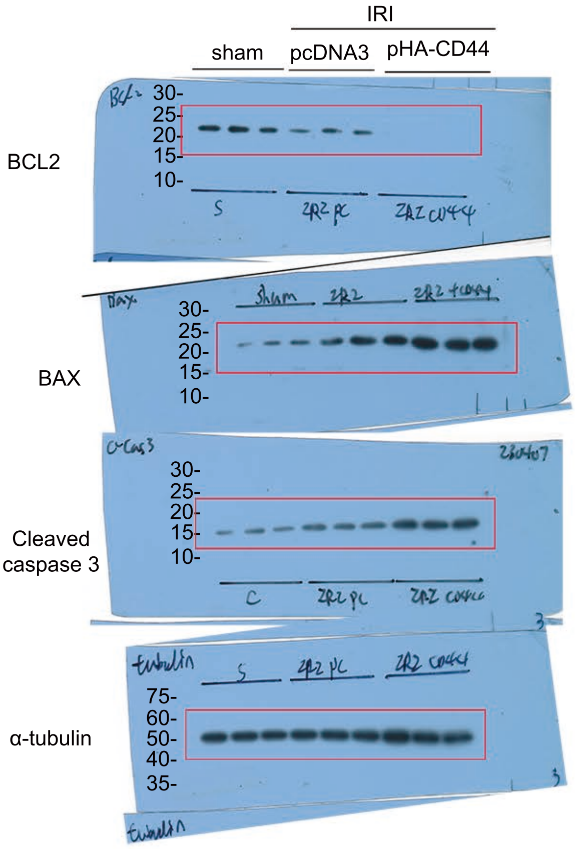

Full unedited gels for Figure 5K

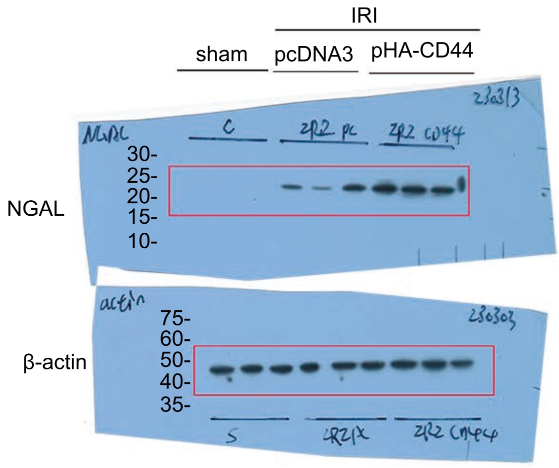

Full unedited gels for Figure 6B

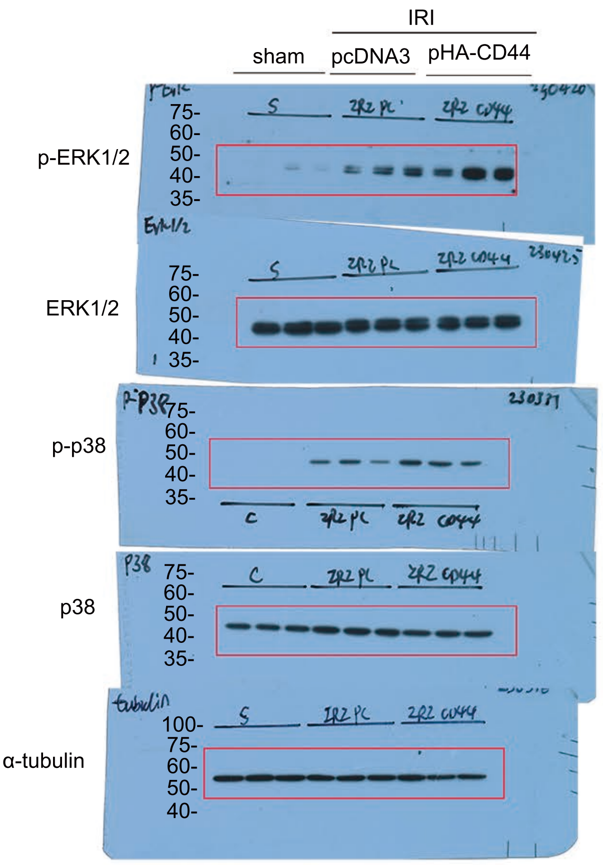

Full unedited gels for Figure 6E

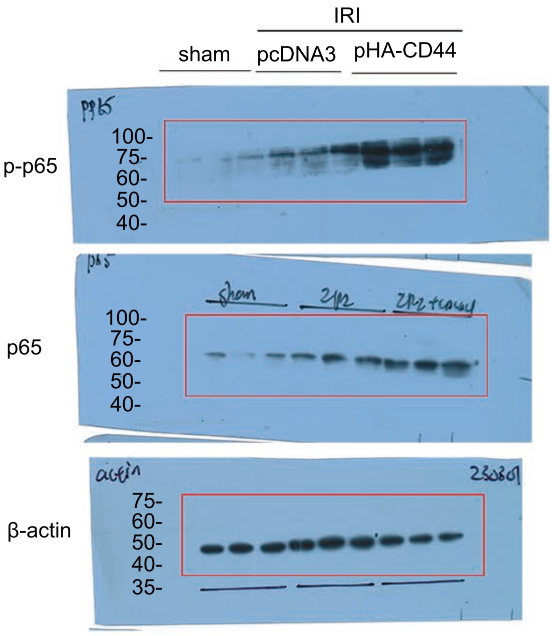

Full unedited gels for Figure 6H

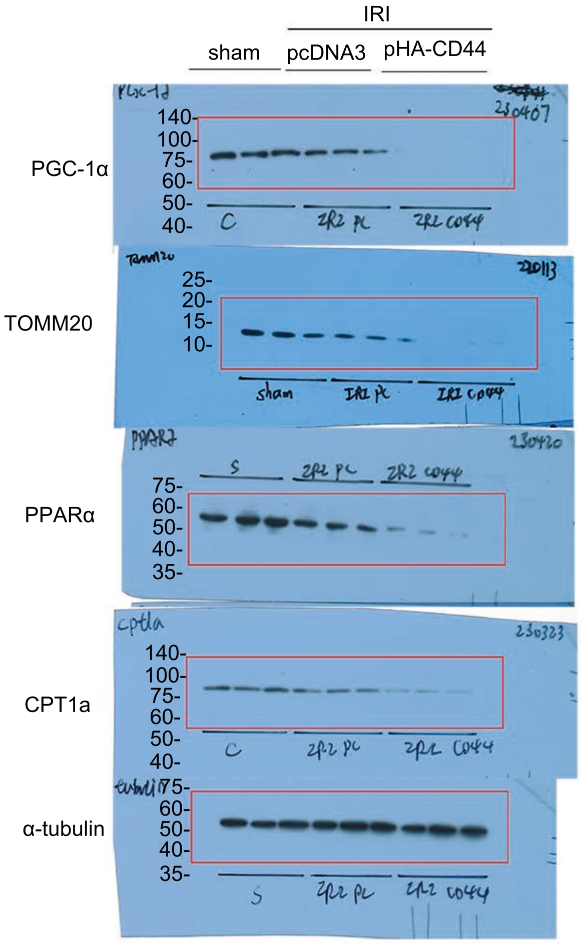

Full unedited gels for Figure 7A

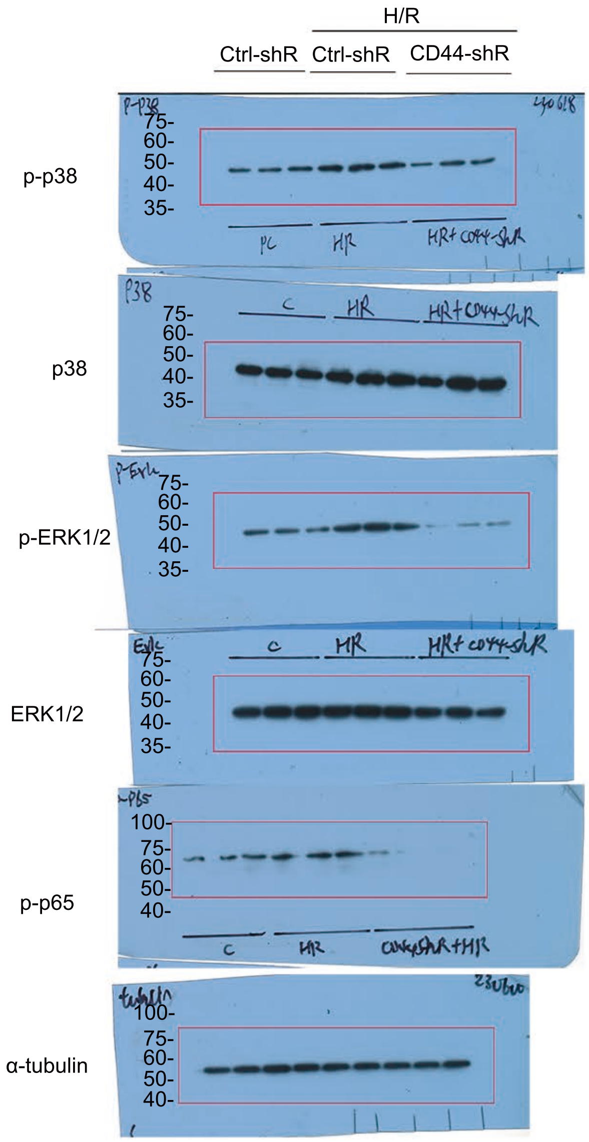

Full unedited gels for Figure 7E

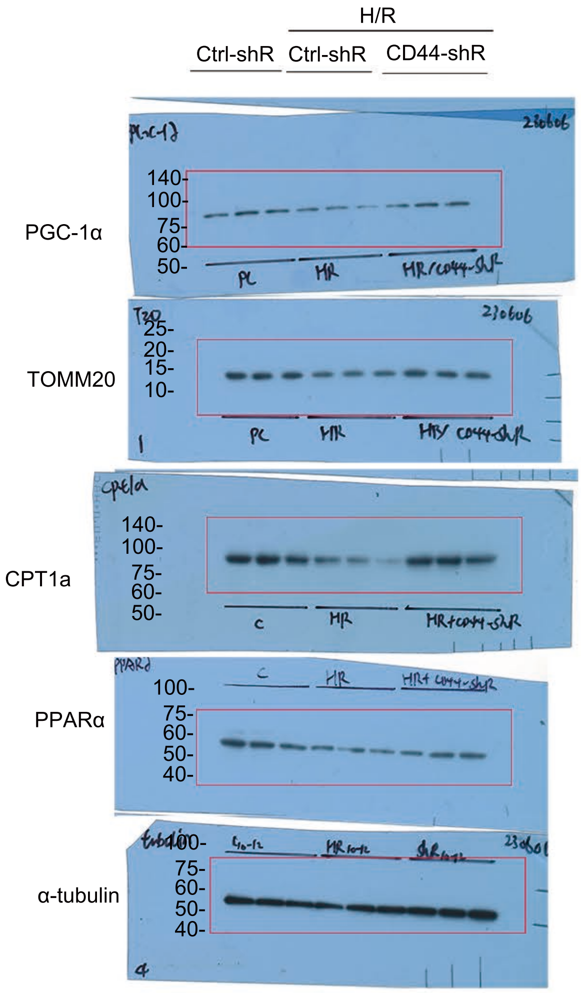

Full unedited gels for Figure 7I

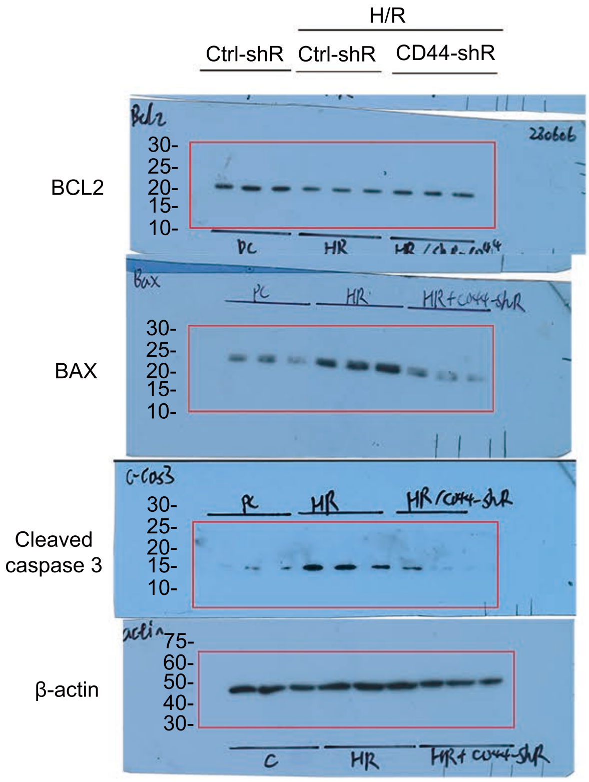

Full unedited gels for Figure 7M

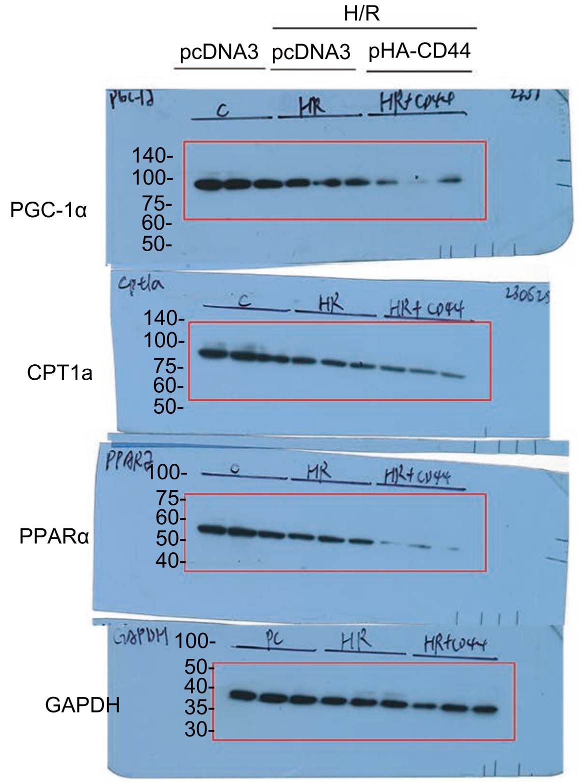

Full unedited gels for Figure 7P

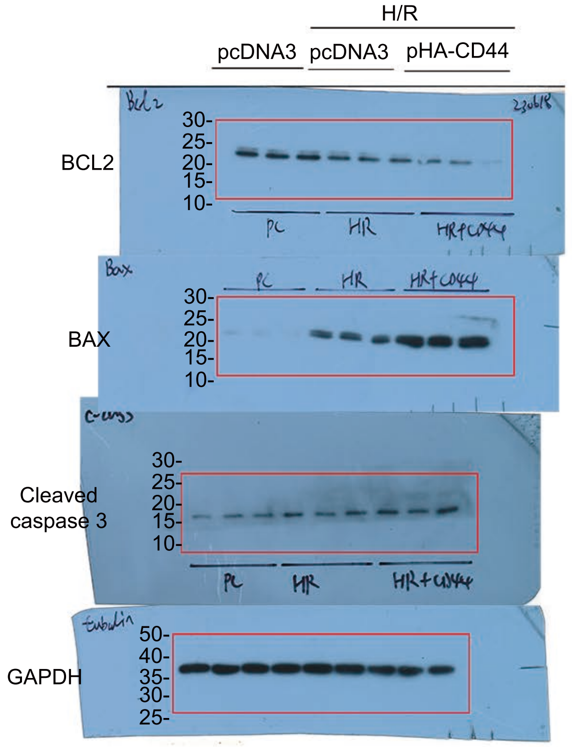

Full unedited gels for Figure 7K

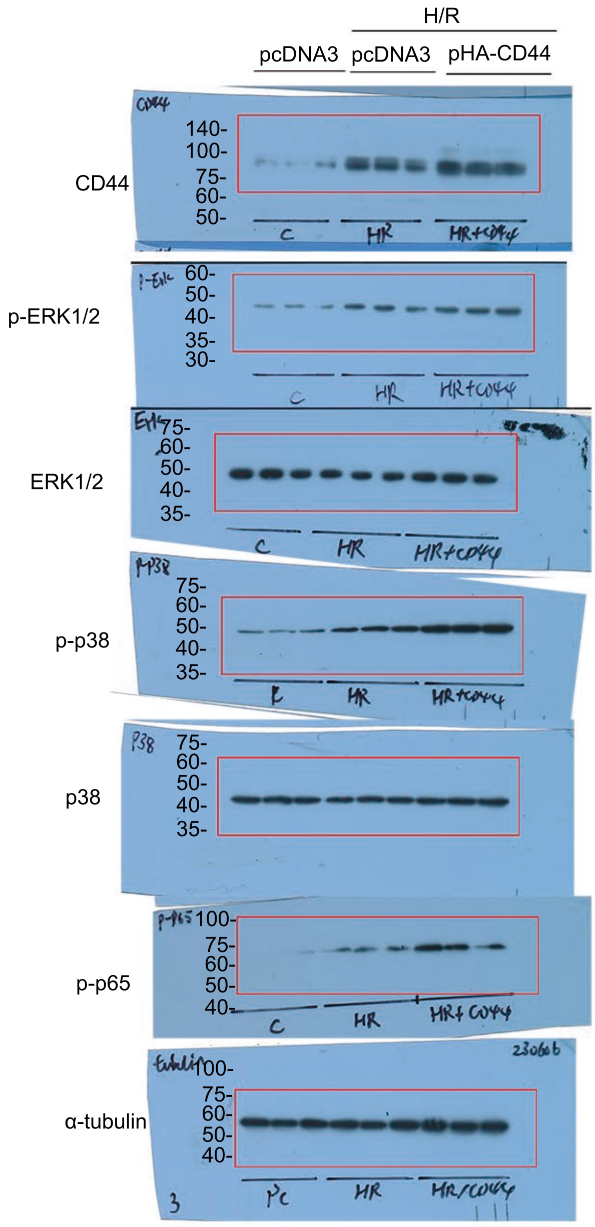

Full unedited gels for Figure 8A

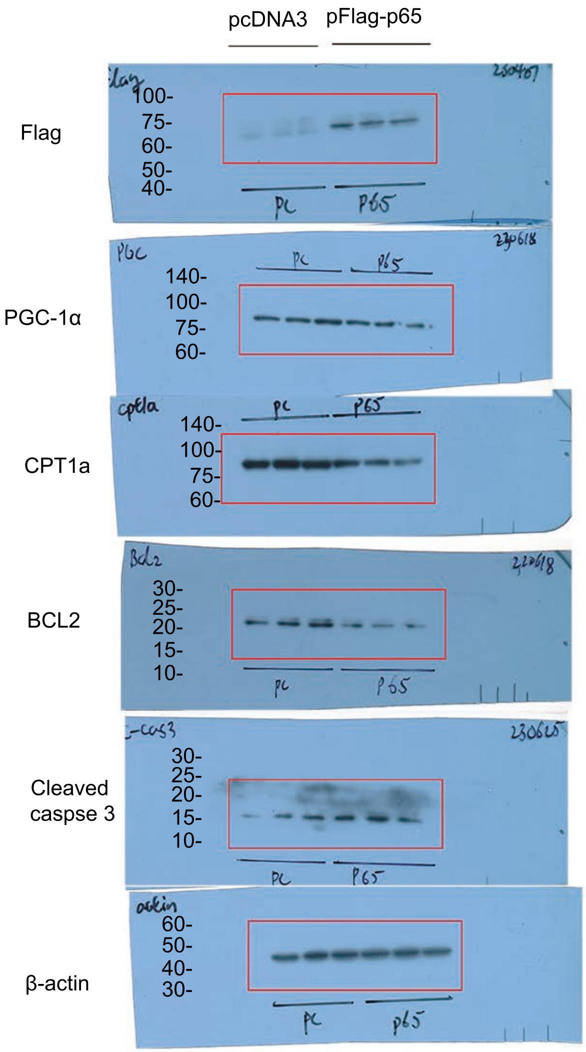

Full unedited gels for Figure 8E

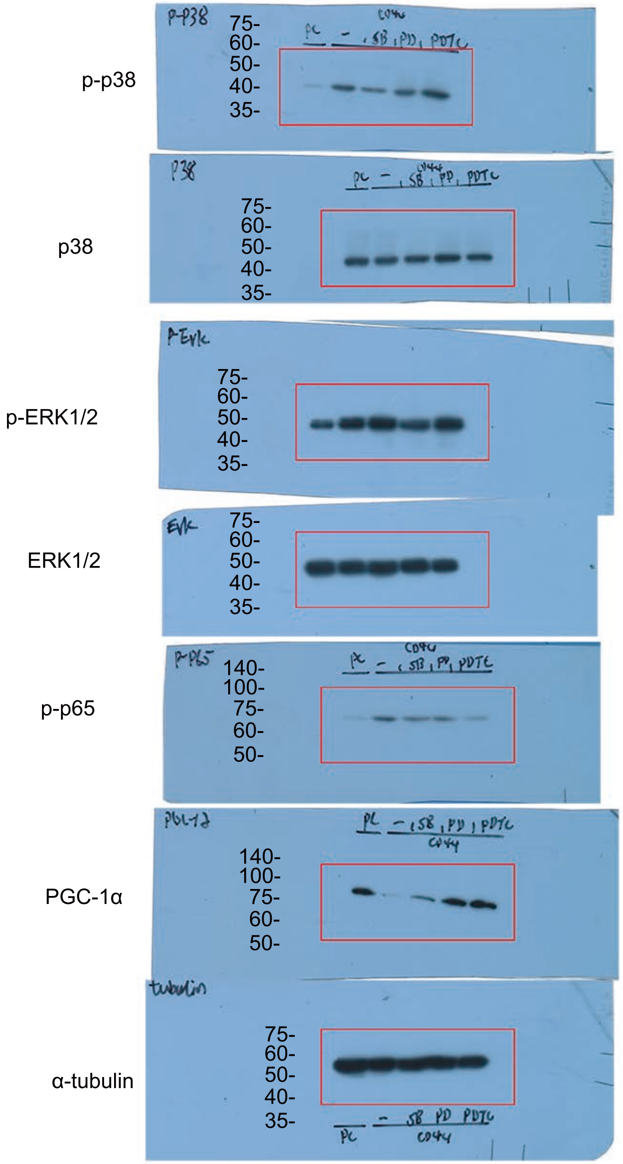

Full unedited gels for Figure 8D

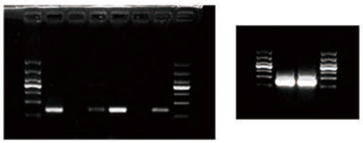

Full unedited gels for Supplementary figure S1A

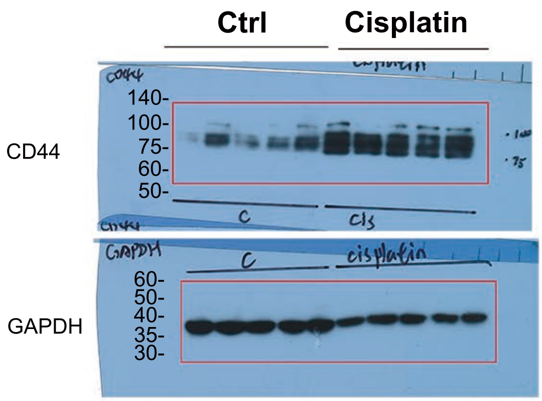

Full unedited gels for Supplementary figure S1D

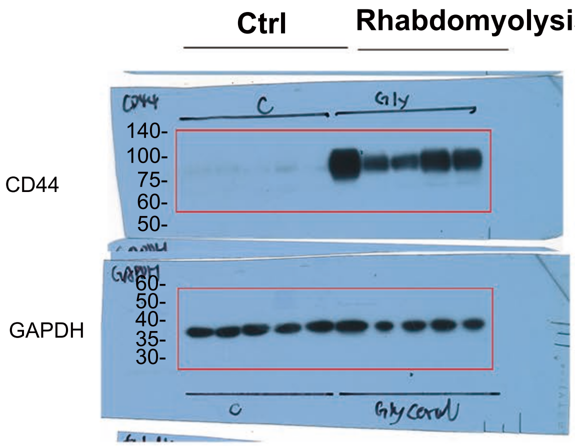

Full unedited gels for Supplementary figure S1J

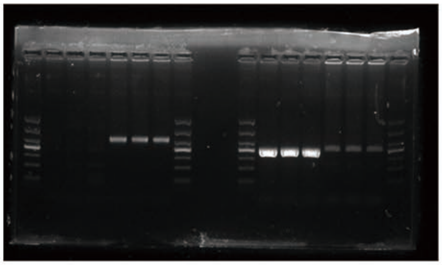

Full unedited gels for Supplementary figure S1L

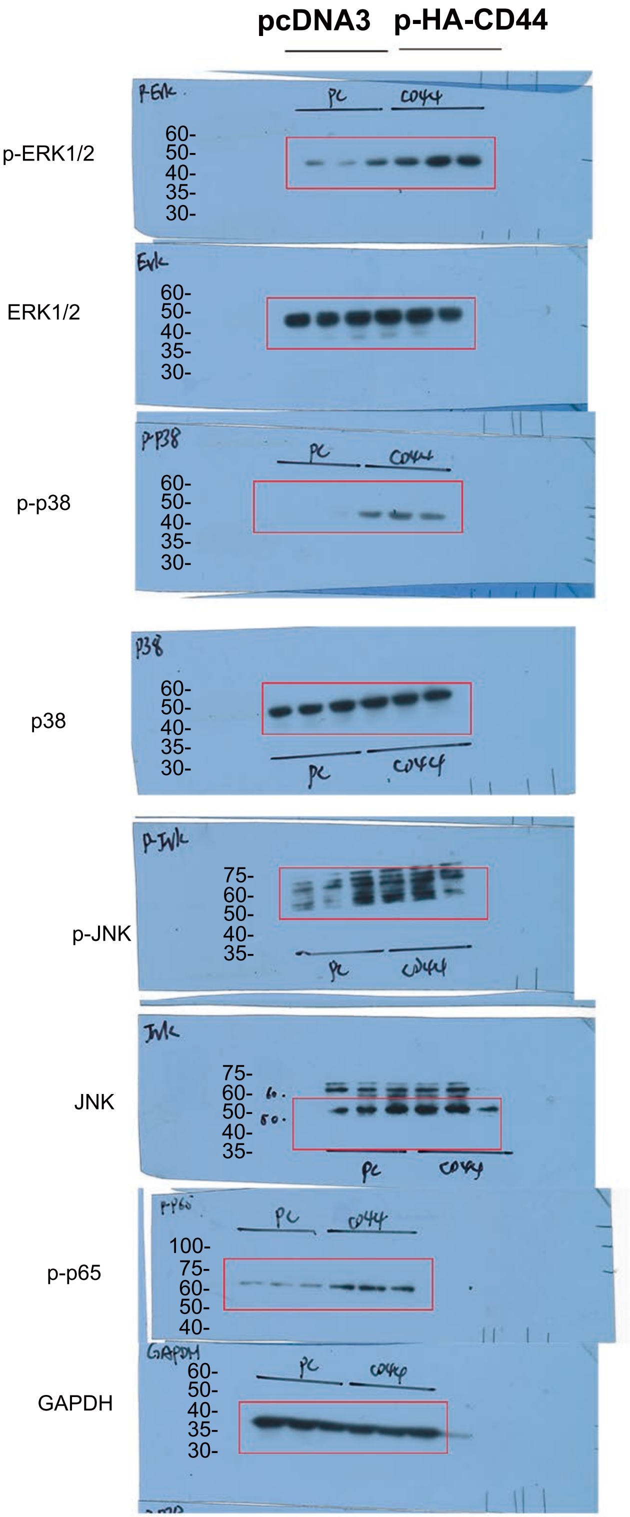

Full unedited gels for Supplementary figure S2B

TOMM20

GAPDH

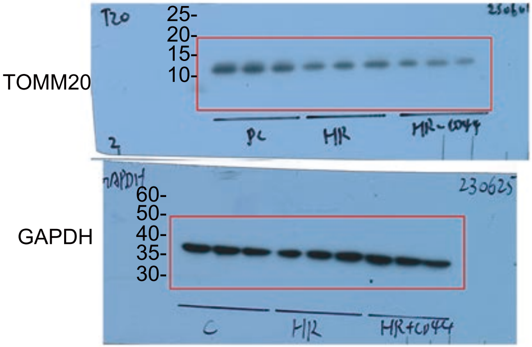

Supplement: Supplementary file 5 — Original Data File [file 41419_2025_7438_MOESM5_ESM.pdf]
